# Novel AU-rich proximal UTR sequences (APS) enhance CXCL8 synthesis upon the induction of rpS6 phosphorylation

**DOI:** 10.1371/journal.pgen.1008077

**Published:** 2019-04-10

**Authors:** Zhiwei Ang, Ricky Abdi Gunawan Koean, Jun Zhi Er, Li Ting Lee, John Kit Chung Tam, Huili Guo, Jeak Ling Ding

**Affiliations:** 1 Department of Biological Sciences, National University of Singapore, Singapore; 2 Institute of Molecular and Cell Biology, A*STAR Institute, Singapore; 3 Department of Surgery, National University of Singapore, 1E Kent Ridge Road, Singapore; Hebrew Univiversity-Hadassah Medical School, IMRIC, ISRAEL

## Abstract

The role of ribosomal protein S6 (rpS6) phosphorylation in mRNA translation remains poorly understood. Here, we reveal a potential role in modulating the translation rate of chemokine (C-X-C motif) ligand 8 (*CXCL8* or Interleukin 8, *IL8*). We observed that more CXCL8 protein was being secreted from less *CXCL8* mRNA in primary macrophages and macrophage-like HL-60 cells relative to other cell types. This correlated with an increase in *CXCL8* polyribosome association, suggesting an increase in the rate of *CXCL8* translation in macrophages. The cell type-specific expression levels were replicated by a *CXCL8-* UTR-reporter (Nanoluc reporter flanked by the 5’ and 3’ UTR of *CXCL8*). Mutations of the *CXCL8*-UTR-reporter revealed that cell type-specific expression required: 1) a 3’ UTR of at least three hundred bases; and 2) an AU base content that exceeds fifty percent in the first hundred bases of the 3’ UTR immediately after the stop codon, which we dub AU-rich proximal UTR sequences (APS). The 5’ UTR of *CXCL8* enhanced expression at the protein level and conferred cell type-specific expression when paired with a 3’ UTR. A search for other APS-positive mRNAs uncovered TNF alpha induced protein 6 (*TNFAIP6*), another mRNA that was translationally upregulated in macrophages. The elevated translation of APS-positive mRNAs in macrophages coincided with elevated rpS6 S235/236 phosphorylation. Both were attenuated by the ERK1/2 signaling inhibitors, U0126 and AZD6244. In A549 cells, rpS6 S235/236 phosphorylation was induced by TAK1, Akt or PKA signaling. This enhanced the translation of the *CXCL8*-UTR-reporters. Thus, we propose that the induction of rpS6 S235/236 phosphorylation enhances the translation of mRNAs that contain APS motifs, such as *CXCL8* and *TNFAIP6*. This may contribute to the role of macrophages as the primary producer of CXCL8, a cytokine that is essential for immune cell recruitment and activation.

## Author summary

Ribosomal protein S6 (rpS6) is a component of the cell translation system. This system produces proteins based on the instructions found on messenger ribonucleic acids or mRNAs. The activity of rpS6 is modified via the attachment of phosphate groups. This rpS6 “phosphorylation” regulates cell size, cell proliferation, glucose homeostasis and cancer defense. However, the molecular mechanism remains poorly understood. Here, we propose that rpS6 phosphorylation may affect the rate of translation of certain mRNAs. These mRNAs contain a high frequency of adenine and uracil bases in the first hundred or so bases of their 3’ UTR sequences, which we dub AU-rich proximal UTR sequences (APS). APS-modulated mRNAs include the immune signaling proteins, *CXCL8* and *TNFAIP6*. This novel mechanism allows macrophages to secrete sufficient CXCL8 protein to attract and activate other immune cells such as neutrophils during injury or disease. It may also be aberrantly upregulated in a subset of tumors, leading to the overexpression of APS-positive genes such as *CXCL8*. Indeed, both rpS6 phosphorylation and *CXCL8* expression are consistently found, by separate studies, to be tumor-promoting and upregulated in cancers. Modulating these APS-positive mRNAs may be a novel strategy to treat diseases.

## Introduction

Translation is an essential step in protein synthesis. Mechanisms that regulate the rate of translation determine the expression levels of a large fraction of the genome. This was revealed by metabolic pulse labeling of global cellular mRNA and protein synthesis rates [1]. Consistently, multiple large-scale transcriptomic and proteomic studies have revealed a lack of correlation between mRNA and protein abundance across different mammalian cell-types and tissues [2,3]. Translational control is mRNA-specific and the specificity is sometimes dependent on sequence motifs within the 5’ untranslated region (UTRs), such as 5′ terminal oligopyrimidine (TOP) [4], which lead to selective protein synthesis during increased activity of eukaryotic translation initiation factor 4E (eIF4E). More recent studies have tentatively proposed that cytosine enriched regulator of translation (CERT) [4] and pyrimidine-rich translational element (PRTE) [5] may also regulate translation. Besides the 5’ UTR, the involvement of the 3’ UTR in conferring translational control has also been hinted [6].

While the translational control mediated by eIF4E is well-studied [5–10], the translational control mediated by phosphorylation of ribosomal protein S6 (rpS6) is still the subject of ongoing investigations. Physiologically, phosphorylation-deficient rpS6 knock-in mice display abnormalities in cell size, cell proliferation, and glucose homeostasis [11]. Aberrant rpS6 phosphorylation has also been implicated in pancreatic tumorigenesis in mice [12,13]. The molecular mechanisms responsible for these physiological effects remain elusive, as rpS6 phosphorylation does not appear to affect global protein synthesis. More recently, rpS6 phosphorylation-deficient transgenic mice [14] were found with impaired translation in a subset of mitochondria-related mRNAs present in neurons [15]. Thus, it appears that rpS6 phosphorylation may alter the translation of a subset of mRNAs, although the exact mechanism and RNA cis-regulatory motifs responsible for the action are unknown.

A possible target for rpS6-mediated translational control is chemokine (C-X-C motif) ligand 8 [*CXCL8* or Interleukin 8, *IL8*]. Studies on *CXCL8* have been complicated by the absence of the *CXCL8* and *CXCR1* gene homologs from the muroid lineage due to a deletion event [16]. The role of CXCL8 in mice appears to have been largely replaced by murine MIP-2 and the murine keratinocyte-derived protein chemokine KC, which activates murine CXCR2 [17,18]. To study CXCL8, researchers have typically relied on clinical observations, *ex vivo* cultures of primary human cells and cell line models. Such methods have revealed a critical role for CXCL8 in the chemotactic recruitment, phagocytosis and degranulation of neutrophils [19]; and in the recruitment and activation of monocytes and lymphocytes during inflammation [20]. CXCL8 induces these functions by activating cell surface receptors, namely CXCR1 and CXCR2 [19]. CXCL8 signaling has also been implicated in a number of diseases including atherosclerosis [21], asthma [22], allergic rhinitis [23] and various cancers [24–26]. In light of the importance of CXCL8, elucidating the mechanism of *CXCL8* translational control may prompt more effective treatments that target these pathways to alleviate CXCL8-mediated diseases.

Here, we investigate if *CXCL8* undergoes translational regulation. While the 5’ UTR of *CXCL8* does not appear to contain TOP [4], CERT [5] or PRTE [6] motifs, certain observations hint at a cell type-specific translation rate. For example, macrophages secrete 70-fold more CXCL8 protein relative to neutrophils despite elevated *CXCL8* mRNA being detected in both cell types [27,28]. This makes macrophages the undisputed primary producer of CXCL8 in the human system. This difference in expression is noteworthy as macrophages and neutrophils are descended from a common myeloid progenitor [29]. These observations suggest that macrophages and neutrophils are prime targets for the study of *CXCL8* translation. To facilitate the acquisition of a large number of cells for *in vitro* studies, our initial experiments were performed with the HL-60 promyeloblast cell line. HL-60 is an established model to study myeloid progenitor cell development [30]. Similar to primary myeloid progenitors [29], HL-60 promyeloblasts can be differentiated into either a macrophage-like (HL-60 macrophage /HL-60 Mac) or polymorphonuclear neutrophil-like (HL-60 PMN) phenotype via PMA or DMSO treatment, respectively [30]. *CXCL8* translation was also studied in KHYG-1, which is an established model of natural killer (NK) cells, and the A549 and NCI-H1299 lung epithelial carcinoma (EC) cells, [31–33]. Using a combination of primary cells and their corresponding cell line models, we elucidated the pathway and *cis*-regulatory RNA motif responsible for the cell type-specific expression of *CXCL8*.

## Results

### CXCL8 protein secretion and polysome association is increased in HL-60 macrophages

**Fig 1:**
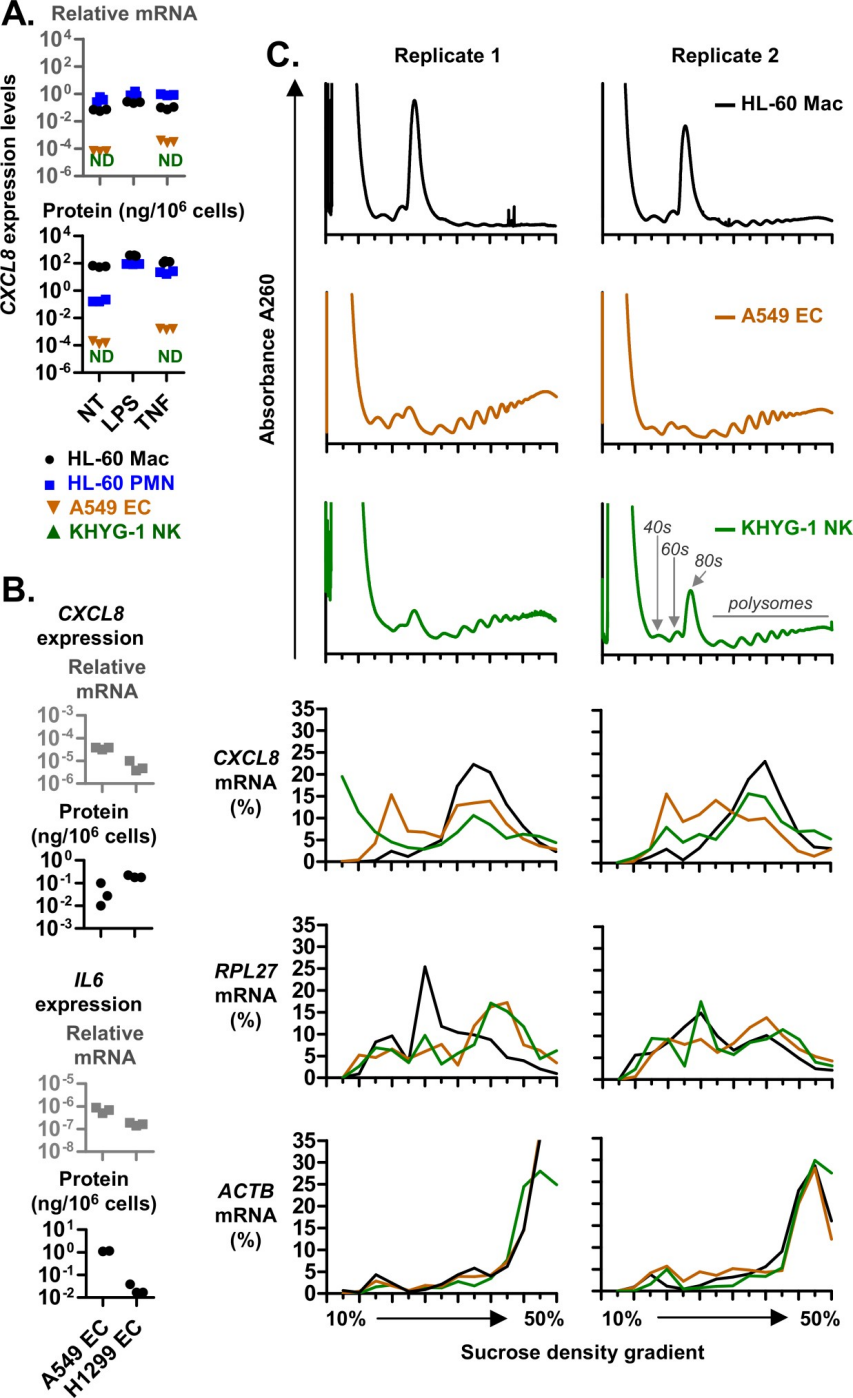
*CXCL8* translation and expression is elevated in HL-60 macrophages. **(A and B)** *CXCL8* and *IL6* expression levels in HL-60 macrophages (HL-60 Mac), neutrophil-like HL-60 polymorphonuclear leukocytes (HL-60 PMNs), A549 and H1299 lung epithelial carcinoma cells (A549 ECs), and KHYG-1 NK cells. Expression was measured at resting state (not treated, NT) or after overnight activation with 100 ng/mL LPS or 10 ng/mL TNF. Protein levels were determined via ELISA. The mRNA levels were determined via real-time PCR and presented as the ratio of *IL6* or *CXCL8* divided by the internal control genes, *RPL27*. **(C)** Polysome profiles of HL-60 Mac, A549 EC and KHYG-1 NK cells were obtained from a continuous sucrose density gradient (left to right: 10–50% sucrose). Compared to A549 EC and KHYG-1 NK cells, a lower proportion of the global mRNA (detected at A260) of HL-60 Mac was detected (at A260) in the high sucrose density fractions associated with polysomes. This is indicative of a low translation rate which may be due to the tendency of HL-60 cells to clump upon differentiation into macrophage (S1B Fig). From 14 fractions spanning the entire sucrose gradient, the levels of specific mRNAs (*CXCL8*, *RPL27* and *ACTB*) were quantified via real-time PCR and presented as a percentage of the sum of all fractions. **(A to C)** Each graph symbol (squares, inverted-triangles or circles) is the result of a replicate experiment. Replicate experiments were performed on different days. ND: Not detected.

First, we compared the expression levels of CXCL8 between HL-60 macrophage (HL-60 Mac), HL-60 neutrophils (HL-60 PMN), A549 EC and KHYG-1 NK cells. We found that HL-60 Mac cells secreted more CXCL8 protein from less *CXCL8* mRNA at the basal level and after stimulation with LPS or TNF (Fig 1A). An increase in secreted CXCL8 protein was also observed from H1299 cells relative to A549 cells despite lower *CXCL8* mRNA levels in H1299 cells (Fig 1B). This increase was not observed for IL6 protein secretion, which mirrored *IL6* mRNA levels and was reduced in H1299 cells relative to A549 cells. We also observed an increase in the percentage of *CXCL8* mRNAs associated with the larger polysomes (towards the right of the graph) in HL-60 Mac relative to A549 EC and KHYG-1 NK cells (Fig 1C). The same increase was not observed for the control mRNAs, *RPL27* and *ACTB*. Since a polysome (or polyribosome) is formed when an mRNA molecule complexes with two or more ribosomes during the translation process, the increased polysome association of *CXCL8* in Hl-60 Mac may be due to an increased rate of translation. Altogether, these observations suggest that the rate of *CXCL8* translation is cell type-specific, leading to elevated CXCL8 protein secretion in macrophages.

### The elevated *CXCL8* expression in HL-60 macrophages required the untranslated regions (UTRs)

**Fig 2:**
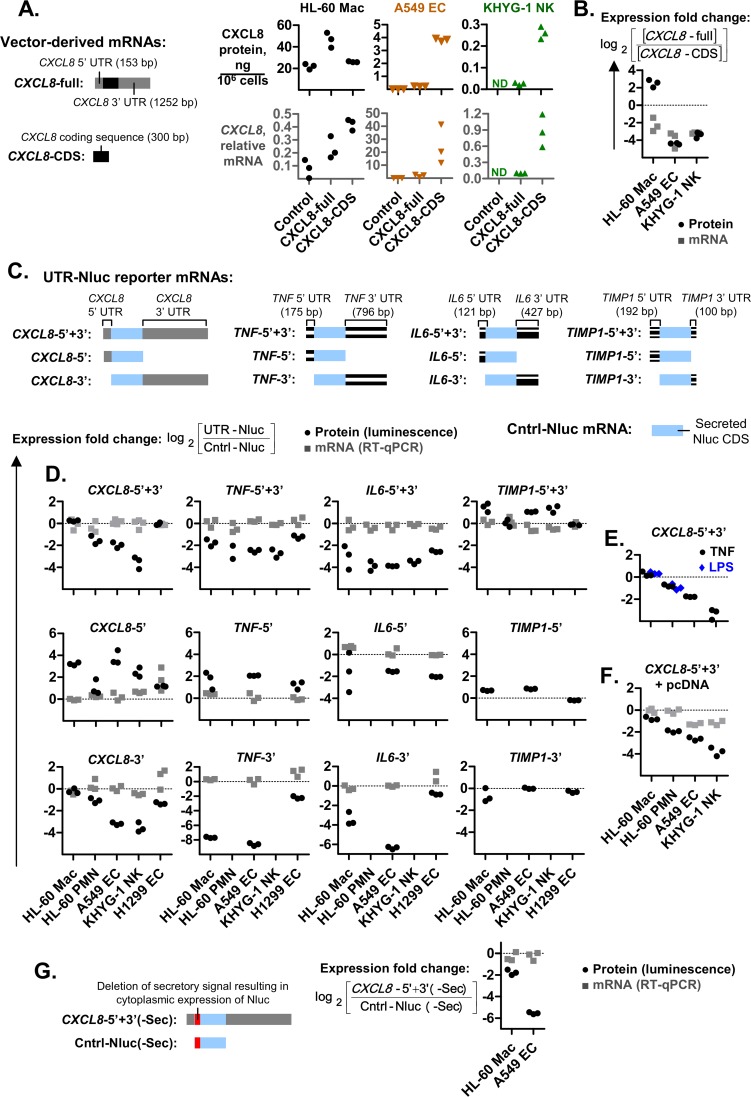
The elevated expression of *CXCL8* in macrophages is mediated by its 3’ UTR. **(A)** *CXCL8* expression in cells transfected with the pcDNA control, *CXCL8*-full or *CXCL8*-CDS plasmids. *CXCL8*-full expresses the full *CXCL8* transcript while *CXCL8*-CDS expresses a mutant with the 5’ and 3’ UTRs deleted. CXCL8 protein levels were determined via ELISA. *CXCL8* mRNA levels were determined via real-time PCR (with primers that prime within the coding sequences) and presented as the ratio of *CXCL8* divided by the internal control gene, *RPL27*. **(B)** Cells transfected with the control plasmid were assumed to only express endogenous *CXCL8*. The *CXCL8* expression observed for these control cells were subtracted from *CXCL8*-full and *CXCL8*-CDS to obtain the expression level of plasmid-derived *CXCL8*, as represented by [*CXCL8*-full] and [*CXCL8*-CDS]. These values were used to calculate the “CXCL8 fold change” in expression based on the mathematical expression shown. **(C, D, E and F)** Schematic representations of the coding sequence (CDS) of secreted Nanoluc (Nluc) fused to the 5’ and 3’ UTRs of *CXCL8*, *TNF*, *IL6* and *TIMP1*.The expression plasmids for these UTR-Nluc reporters were transfected into parallel cell cultures. The resulting Nluc protein and mRNA expression levels were quantified via luciferase assay and real-time PCR, respectively, after overnight incubation. The ratio of UTR-Nluc over Cntrl-Nluc expression was then determined and presented as the log_2_ fold change in Nluc expression. Nluc mRNA levels were standardized to the NeoR gene expressed from the pcDNA3.1(+), which is the expression plasmid for the UTR- and Cntrl-Nluc constructs. Cells were treated with 100 ng/mL LPS or 10 ng/mL TNF, 3 h after transfection in panel E. The amount of UTR- and Cntrl-Nluc plasmid transfected was halved and replaced with empty pcDNA3.1 plasmid in panel E. **(G)** Mutant variants of the *CXCL8*-5’+3’ UTR-Nluc reporter and Cntrl-Nluc mRNAs, with deletions in the secretory peptide signals resulting in cytoplasmic expression of Nluc. **(A to G)** Each graph symbol (squares, triangles, inverted-triangles or circles) is the result of a replicate experiment. Replicate experiments were performed on different days.

We hypothesized that the increased *CXCL8* expression observed in HL-60 Mac (Fig 1A) may involve the 5’ and 3’ UTR sequences. To investigate the role of the UTRs, we generated expression vectors containing the *CXCL8* coding sequence alone (*CXCL8*-CDS) and the full *CXCL8* mRNA sequence which includes the 5’ and 3’ UTRs (*CXCL8*-full) (Fig 2A). These constructs were transfected into the HL-60 Mac, A549 EC and KHYG-1 NK cells. These cell lines could be efficiently transfected (S1A Fig) and upon transfection with the *CXCL8*-full plasmid displayed an increase in *CXCL8* mRNA levels relative to the empty plasmid controls (Fig 2A). A proportional increase in CXCL8 protein was also observed. This suggests that vector-derived “*CXCL8*-full” was being expressed at the same rate as the endogenous *CXCL8* found in the controls.

We then determined the effect of the UTRs on *CXCL8* expression by calculating the fold change of *CXCL8* mRNA and CXCL8 protein in *CXCL8*-full transfected cells relative to *CXCL8*-CDS. The 3’ UTR of *CXCL8* reportedly contains adenylate-uridylate-rich elements (AREs) which accelerated mRNA degradation [34–36]. These AREs are present in *CXCL8*-full but absent from the UTR-deficient *CXCL8*-CDS construct. This may explain why *CXCL8*-full transfections displayed *CXCL8* mRNA that were 4- to 16-fold lower than *CXCL8*-CDS in all the cell types tested (Fig 2B). For A549 EC and KHYG-1 NK cells, this coincided with 8- to 16-fold reductions in CXCL8 protein. For HL-60 Mac by contrast, a 4-fold increase in CXCL8 protein levels was observed. This suggests that more CXCL8 protein was being produced and secreted from less *CXCL8* mRNA in HL-60 Mac relative to A549 and KHYG-1 cells. Overall, these findings suggest that the elevated *CXCL8* expression in macrophages (Fig 1) is reproduced by vector-derived *CXCL8* mRNA and requires the UTRs (Fig 2A and 2B).

### The 3’ UTR of *CXCL8* conferred cell type-specific expression

To determine if the *CXCL8* UTR sequences alone were sufficient to confer enhanced expression in HL-60 Mac, we generated Nluc reporters with UTR fusions (UTR-Nluc) (Fig 2C). The Nluc reporter allowed us to study a much wider range of hard-to-transfect cells including primary macrophages, NK cells and HL-60 PMNs, since even low levels of vector-derived Nluc can be accurately quantified due to a lack of endogenous Nluc expression. The effect of the fused UTRs on Nluc expression in different cell types was calculated as the fold change of UTR-Nluc relative to Cntrl-Nluc (Nluc reporter with no UTR fusions) (Fig 2D).

*CXCL8*-5’+3’ (Nluc reporter with CXCL8 5’ and 3’ UTR sequences) expression was compared between HL-60 Mac, HL-60 PMNs, A549 ECs, KHYG-1 NK and H1299 ECs (Fig 2D). *CXCL8*-5’+3’ protein levels were elevated in HL-60 Mac and H1299 ECs. This was not due to differences in expression at the mRNA level, which remained largely unchanged among all cell types. This is consistent with an elevated rate of *CXCL8*-5’+3’ translation in HL-60 Mac and H1299 EC cells. The cell type-specific expression of *CXCL8*-5’+3’ was not noticeably altered by LPS or TNF treatments (Fig 2E) or by halving the amount of UTR- and Cntrl-Nluc plasmid transfected and replacing the other half with empty pcDNA3.1 plasmid (Fig 2F). We also deleted the Nluc secretory signal from *CXCL8*-5’+3’ and Cntrl-Nluc, resulting in cytoplasmic expression of Nluc. *CXCL8*-5’+3’(-Sec) protein levels remained elevated in HL-60 mac relative to A549 ECs (Fig 2G). This suggests that the cell type-specific differences were not due to differences in protein secretion. Overall, these findings suggest that the *CXCL8* UTR sequences contribute to its elevated expression in HL-60 Mac and H1299 cells.

The cell type-specific Nluc protein fold changes observed for *CXCL8*-5’+3’ was also distinct from that observed for *TNF*-5’+3’, *IL6*-5’+3’ and *TIMP1*-5’+3’ (Fig 2C and 2D). Relative to Cntrl-Nluc, both *TNF*-5’+3’ and *IL6*-5’+3’ reduced Nluc protein expression across all cell lines. This is consistent with previous reports that the 3’ UTR sequences of *TNF* and *IL6* contain AREs that reduce expression [37,38]. By contrast, the protein and mRNA levels of *TIMP1*-5’+3’ were not substantially different from Cntrl-Nluc, since the *TIMP1* UTRs are not known to contain any AREs or other functional sequences. Altogether, these results suggest that the *CXCL8* UTRs mediate a cell type-specific expression pattern, at the protein level, that is not observed for *TNF*, *IL6* and *TIMP-1*.

The UTR-Nluc reporter constructs tested thus far contained both the 5’ and 3’ UTRs. We also tested constructs containing either the 5’ or 3’ UTR alone (*CXCL8*-5’ and *CXCL8*-3’) to determine their individual contributions (Fig 2C and 2D). The Nluc protein and mRNA fold changes of *CXCL8*-3’ largely followed the pattern observed for *CXCL8*-5’+3’. This suggests that the cell type-specific expression of *CXCL8* is largely mediated by its 3’ UTR.

### A conserved motif within the 5’ UTR of *CXCL8* enhanced protein expression

**Fig 3:**
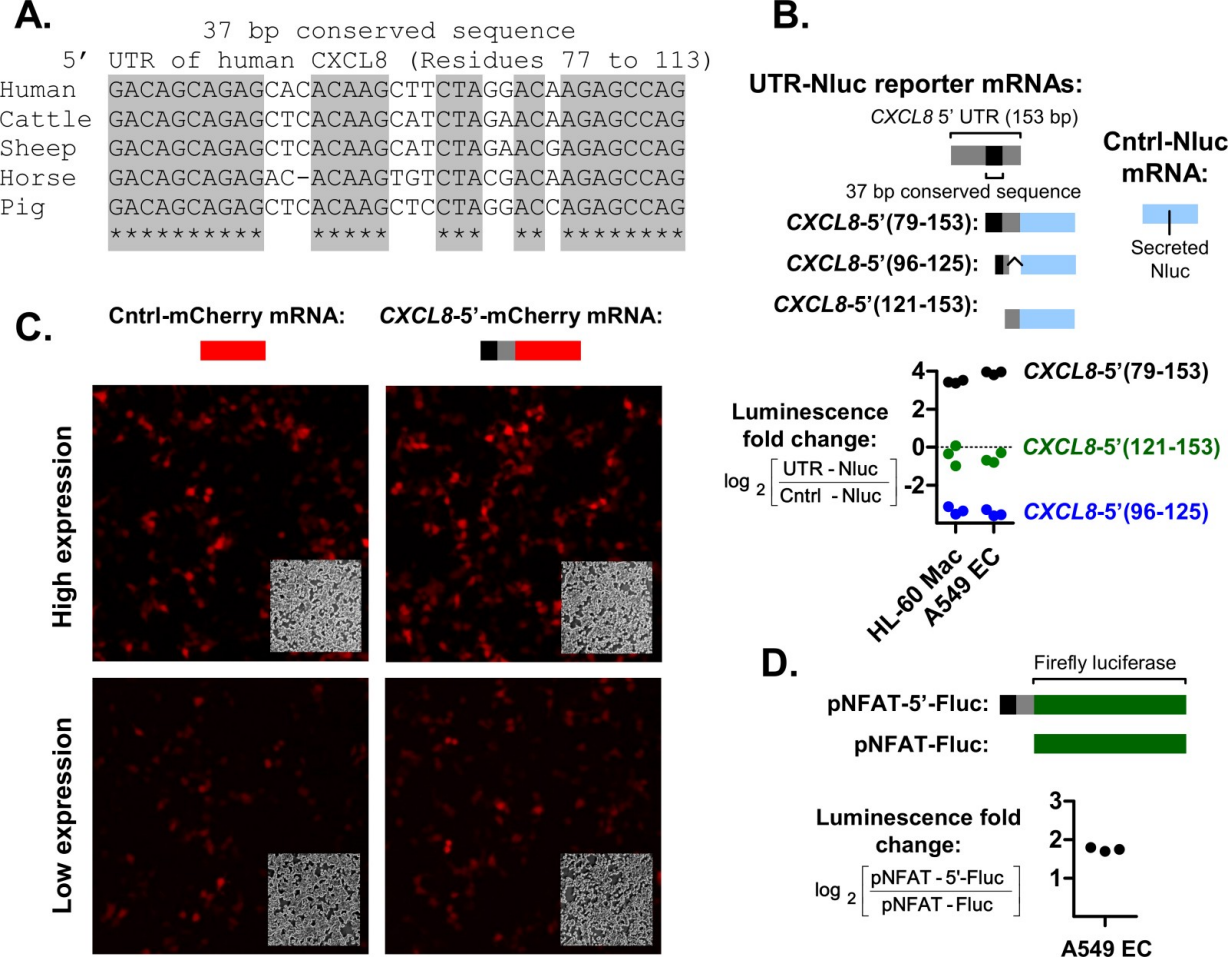
A 37-bp conserved sequence within the 5’ UTR of *CXCL8* enhances expression. **(A)** A 37-bp sequence found within the 5’ UTR of *CXCL8* is conserved across the homologs of multiple species. **(B)** Schematic representations of UTR-Nluc reporters with *CXCL8* 5’ UTR deletions are shown. The expression plasmids for the UTR-Nluc reporters were transfected into parallel cell cultures. The resulting Nluc protein expression levels were quantified via luciferase assay after overnight incubation. The ratio of UTR-Nluc over Cntrl-Nluc expression was then determined and presented as a log_2_ value. **(C and D)** The truncated 5’ UTR of CXCL8 containing the conserved 37 bp sequence was also fused mCherry red fluorescent protein and firefly lucifease Fluc to form the *CXCL8*-5’-mCherry and pNFAT-5’-Fluc reporters. Both reporter proteins were expressed into the cytoplasm. Fluc is additionally under the control of a NFAT promoter, which is weaker compared to the CMV promoter upstream of the Nluc and mCherry reporters. Fluorescence micrographs of Cntrl-mCherry and *CXCL8*-5’-mCherry expression in A549 ECs and the corresponding phase contrast images displayed at the bottom right corner of each fluorescence micrograph are shown in panel C. The ratio of pNFAT-5’-Fluc over pNFAT-Fluc expression, presented as a log_2_ value, is shown in panel D. Each graph symbol (squares) is the result of a replicate experiment. Replicate experiments were performed on different days.

*CXCL8*-5’ protein levels were increased relative to Cntrl-Nluc in all cell lines (positive log_2_ fold change values) (Fig 2D). By multiple sequence alignment, we found a conserved 37-bp motif within the 5’ UTR of *CXCL8* (Fig 3A), and hypothesized that it was involved in the enhancement. Indeed, the enhanced expression was retained when the 5’ UTR was shortened into a 75-bp fragment [CXCL8-5’(79–153)] containing the conserved 37-bp motif; and was abolished upon further shortening to the CXCL8-5’(96–125) and CXCL8-5’(121–153) fragments (Fig 3B). The *CXCL8* 5’ UTR fragment also successfully enhanced mCherry [*CXCL8*-5’-mCherry] (Fig 3C) and firefly luciferase (pNFAT-5’-Fluc) expression (Fig 3D), confirming that the enhanced expression was applicable to other genes and was not specific to Nluc. The expression enhancement also occurred when driven by the NFAT promoter (i.e. in pNFAT-5’-Fluc”), which is a weaker promoter relative to the constitutive CMV promoter used for *CXCL8*-5’(79–153) and *CXCL8*-5’-mCherry. In summary, our findings demonstrate that a conserved 75-bp sequence motif within the 5’ UTR of *CXCL8* enhances expression.

### The cell type-specific expression is independent of known ARE motifs

The reduced mRNA levels of *CXCL8*-full relative to *CXCL8*-CDS (Fig 2A and 2B) are consistent with previous reports of increased *CXCL8* mRNA degradation due to AREs within its 3’ UTR [34–36]. Surprisingly, this reduction was not observed for *CXCL8*-5’+3’ relative to Cntrl-Nluc (Fig 2B), which may be due to the much higher rate of mRNA transcription masking the reduction from mRNA degradation. Accordingly, when the rate of *CXCL8*-5’+3’ transcription was presumably reduced (by halving the amount of UTR- and control-Nluc vector transfected and replacing the other half with empty pcDNA vector), we observed a reduction in *CXCL8*-5’+3’ mRNA levels relative to Cntrl-Nluc mRNA in A549 ECs and KHYG-1 NK cells (Fig 2F).

**Fig 4:**
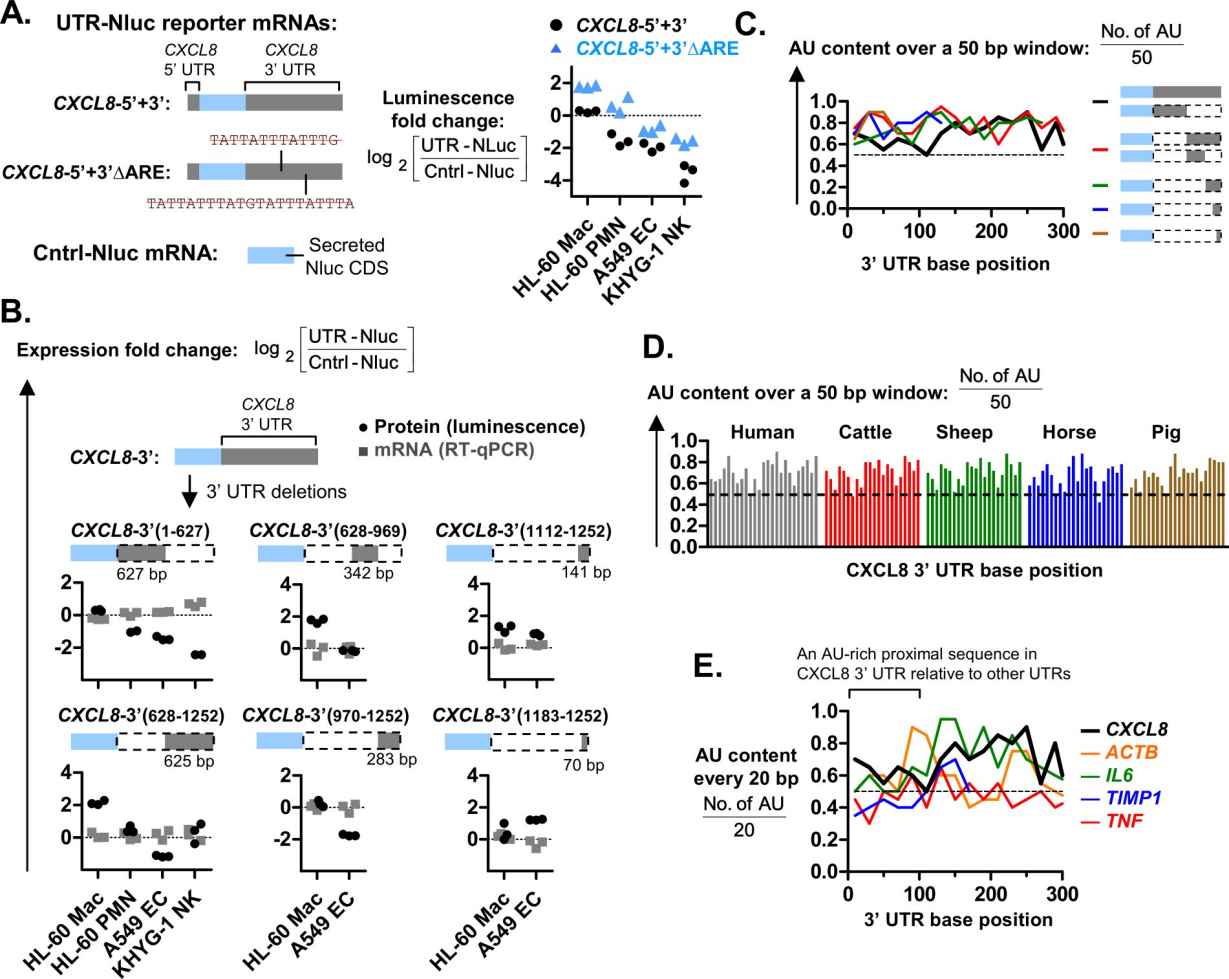
The 3’ UTRs of *CXCL8* contains AU-rich proximal sequences (APSs). **(A and B)** Schematic representations of UTR-Nluc reporters with CXCL8 3’ UTR deletions are shown. The *CXCL8*-5’+3’ΔARE mutant was generated by deleting known AU-rich elements (AREs) from *CXCL8*-5’+3’, as shown with red “strikeouts”. The expression plasmids for the UTR-Nluc reporter mRNAs were transfected into parallel cell cultures. The resulting Nluc protein and mRNA expression levels were quantified via luciferase assay and real-time PCR, respectively, after overnight incubation. The ratio of UTR-Nluc over Cntrl-Nluc expression was then determined and presented as log_2_ values. Each graph symbol (squares, triangles, or circles) is the result of a replicate experiment. Replicate experiments were performed on different days. **(C)** Elevated adenine and uracil base content (AU content) in the 3’ UTR of the UTR-Nluc reporters with *CXCL8* 3’ UTR deletions. **(D)** The elevated AU content of the CXCL8 3’ UTR is conserved in mammals. **(E)** Elevated AU content in the first 100 bases (proximal end) of the *CXCL8* 3’ UTR relative to the *ACTB*, *TNF*, *IL6* and *TIMP1* 3’UTRs.

The ARE motifs reportedly also reduced *CXCL8* expression [34–36]. However, when we deleted two AREs (predicted by RegRNA2.0 [39]) within the 3’ UTR of *CXCL8*, the resulting *CXCL8*-5’+3’ΔARE mutant construct retained the cell type-specific expression observed for *CXCL8*-5’+3’, albeit with increased expression across all cell types (Fig 4A). This suggests that the ARE motifs reduce CXCL8 expression equally across all cell-types and are not responsible for the cell type-specific expression.

### *CXCL8* 3’ UTR fragments of at least 300 bp conferred cell type-specific expression

To identify the *CXCL8* 3’ UTR sequences that are involved in the cell type-specific expression, we split the 3’ UTR and cloned the halves into the *CXCL8*-3’(1–627) and *CXCL8*-3’(628–1252) UTR-Nluc reporters (Fig 4B). Both reporters retained increased Nluc protein levels in HL-60 Mac relative to HL-60 PMN, A549 ECs and KHYG-1 NK cells. The 3’ UTR was split further into fragments of around 300 bp in length, generating the *CXCL8*-3’(628–969) and *CXCL8*-3’(970–1252) reporters. These UTR-Nluc reporters continued to display increased Nluc protein levels in HL-60 Mac relative to A549 ECs. The cell type-specific protein levels were eventually lost upon further deletion into a 141-bp and 70-bp fragment [*CXCL8*-3’(1112–1252’) and *CXCL8*-3’(1183–1252) respectively]. Taken together, these findings suggest that all the *CXCL8* UTR fragments of at least 300 bp confer cell type-specific expression.

### All 3’ UTRs that mediate cell type-specific expression also contain AU-rich proximal UTR sequences (APS)

All the *CXCL8* 3’ UTR fragments that have conferred increased Nluc protein levels in HL-60 Mac relative to A549 ECs, have a high adenylate-uridylate or AU content in their 3’ UTR sequences (Fig 4C). Accordingly, this AU-rich sequence feature is also observed in the full-length *CXCL8* 3’ UTR. A high AU content in the first hundred or so bases of the *CXCL8* 3’ UTR, appears to be conserved among the known mammalian homologs of *CXCL8* (Fig 4D). This may indicate a functional role that was maintained by natural selection. A relatively high AU content in the first hundred or so bases of the 3’ UTR also distinguishes *CXCL8* from *TNF*, *IL6* and *TIMP1* (Fig 4E). This may explain why *TNF*-5’+3’, *IL6*-5’+3’ and *TIMP1*-5’+3’ displayed cell type-specific expression that were distinct from *CXCL8*-5’+3’ (Fig 2D). Based on these observations, we hypothesized that a high AU content in the first hundred or so bases of the *CXCL8* 3’ UTR may be the sequence feature responsible for mediating increased protein synthesis in HL-60 Mac. Henceforth, we dub these AU-rich proximal UTR sequences (APS).

### The deletion or insertion the APS modified cell type-specific expression

**Fig 5:**
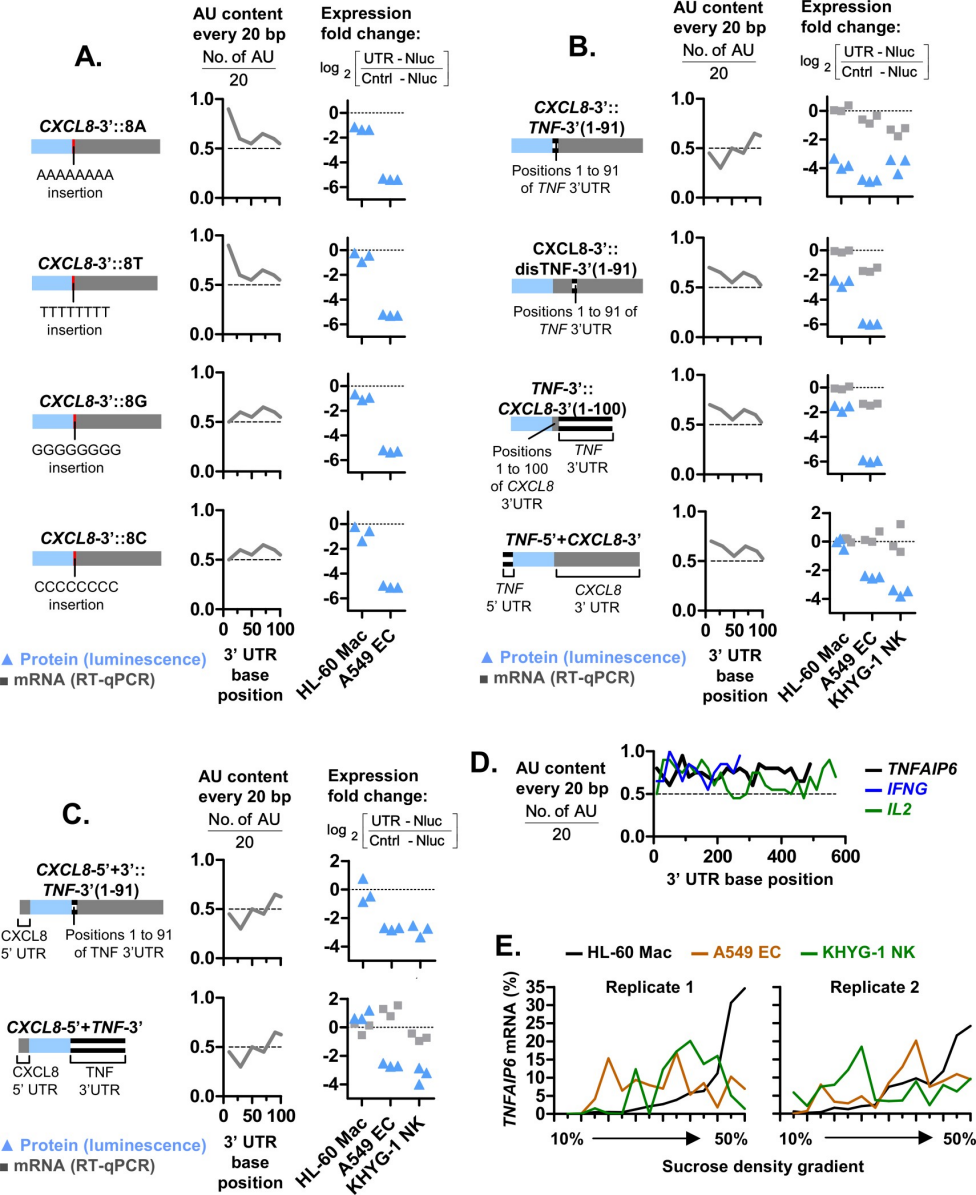
APSs conferred elevated expression in HL-60 macrophages. **(A, B and C)** Schematic representations of UTR-Nluc reporters with UTR mutations are shown. The AU content in the first 100 bases of the 3’ UTR of these UTR-Nluc reporter mutants are shown in line charts. Positions 1 to 91 of the 3’ UTR of TNF was inserted in between positions 285 and 286 of the *CXCL8*-3’ reporter to generate the *CXCL8*-3’::dis*TNF*-3’(1–91) mutant. The expression plasmids for these UTR-Nluc reporter mRNAs were transfected into parallel cell cultures. The resulting Nluc protein and mRNA expression levels were quantified via luciferase assay and real-time PCR, respectively, after overnight incubation. The ratio of UTR-Nluc over Cntrl-Nluc expression was then determined and presented as log_2_ values. Each graph symbol (squares or triangles) is the result of a replicate experiment. Replicate experiments were performed on different days. **(D)** The 3’ UTR of *TNFAIP6*, *IFNG* and *IL2* contain putative APSs. **(E)** From 14 fractions spanning the entire sucrose gradients displayed in Fig 1C, the levels of specific *TNFAIP6* mRNA were quantified via real-time PCR and presented as a percentage of the sum of all fractions. Replicate experiments were performed on different days and the data from 2 replicates are shown.

To investigate the role of the APS, we inserted short sequences into the 3’ UTR to disrupt or introduce the APS-motif. Performing insertions instead of substitutions ensured that the full 3’ UTRs sequences were retained. This ensured that any differences in expression observed were not due to deletions of functional motifs within the 3’ UTR sequences. The presence of the APS was determined by calculating the AU-content of the first 100 bp of the mutated 3’ UTR. We then determined if the protein levels of the mutant UTR reporter was elevated in HL-60 Mac relative to A549 ECs, which is indicative of the cell type-specific expression of CXCL8. The insertion of 8 bases immediately upstream of the 3’ UTR sequence of the *CXCL8*-3’ reporter, did not disrupt the APS and cell type-specific expression (Fig 5A). Only the insertion of a longer sequence, namely the first 91 bp of *TNF* 3’ UTR, resulted in an APS-deficient *CXCL8*-3’::*TNF*-3’(1–91) reporter (Fig 5B). This coincided with the disruption of cell type-specific expression as a much smaller difference in *CXCL8*-3’::*TNF*-3’(1–91) reporter protein levels was observed between HL-60 Mac and A549 ECs. As a control, the first 91 bp of the TNF 3’ UTR was also inserted in between positions 285 and 286 of the 3’ UTR of “CXCL8 3’” (Fig 5B). The resulting “CXCL8 Δ3’-dis” mutant remained APS-positive and displayed elevated protein levels in HL-60 Mac. Inversely, we also inserted the first 100 bp of the *CXCL8* 3’ UTR sequence immediately before the *TNF* 3’ UTR of the APS-deficient *TNF*-3’ reporter (Fig 5B). This generated an APS-positive mutant, *TNF*-3’::*CXCL8*-3’(1–100) which displayed elevated protein levels in HL-60 Mac relative to A549 ECs. Altogether, these data are consistent with the APS-motif conferring cell type-specific expression.

### The *CXCL8* 5’ UTR conferred cell type-specific expression when paired with a 3’ UTR

We had deduced that the cell type-specific expression of CXCL8 was largely mediated by its 3’ UTR. This was based on the observation that the cell type-specific Nluc protein and mRNA fold changes of *CXCL8*-5’+3’ was largely replicated by *CXCL8*-3’ but not *CXCL8*-5’ (Fig 2C and 2D). Indeed, the ability of the *CXCL8* 3’ UTR to mediate enhanced expression in HL-60 Mac relative to A549 ECs occurred even in the presence of the *TNF* 5’ UTR sequence, as was observed for the *TNF*-5’+*CXCL8*-3’ reporter (Fig 5B). However, our subsequent assays revealed that the 5’ UTR of *CXCL8* may not be entirely benign. We observed that the protein levels of *CXCL8*-5’+3’::*TNF*-3’(1–91) and *CXCL8*-5’+*TNF*-3’ were elevated in HL-60 Mac relative to A549 EC, despite being APS-deficient (Fig 5C). This may have been mediated by the presence of the 5’ UTR of CXCL8. The lack of this cell type-specific expression in the *CXCL8*-5’ reporter may have been due to the absence of a 3’ UTR sequence (Fig 2D). Thus, these findings suggest that the 5’ UTR of CXCL8 may confer cell type-specific expression when paired with a 3’ UTR.

### The polysome association of *TNFAIP6*, a putative APS-positive mRNA, was elevated in HL-60 macrophages

With the elucidation of the novel APS-motif, we then investigated if the presence of APS could be used to predict elevated translation in macrophages. An analysis of proximal AU content in the 3’ UTR of common cytokine signaling genes revealed that the *TNFAIP6*, *IFNG* and *IL2* genes possess high AU-contents in the first hundred or so bases of their 3’ UTRs (Fig 5D), which is indicative of the presence of APS. Amongst these genes, only the *TNFAIP6* mRNA was expressed at a detectable level in HL-60 Mac. In agreement with our prediction, the polysome association of *TNFAIP6* was elevated in HL-60 Mac relative to A549 EC and KHYG-1 NK cells. This was indicated by an increase in the percentage of *TNFAIP6* mRNA found in the larger polysomes fractions, towards the right of the graph, in HL-60 Mac (Fig 5E). Thus, our results suggest that *TNFAIP6* may also contain a functional APS-motif.

### The inhibition of ERK1/2 signaling reduced the expression of APS-positive mRNAs

**Fig 6:**
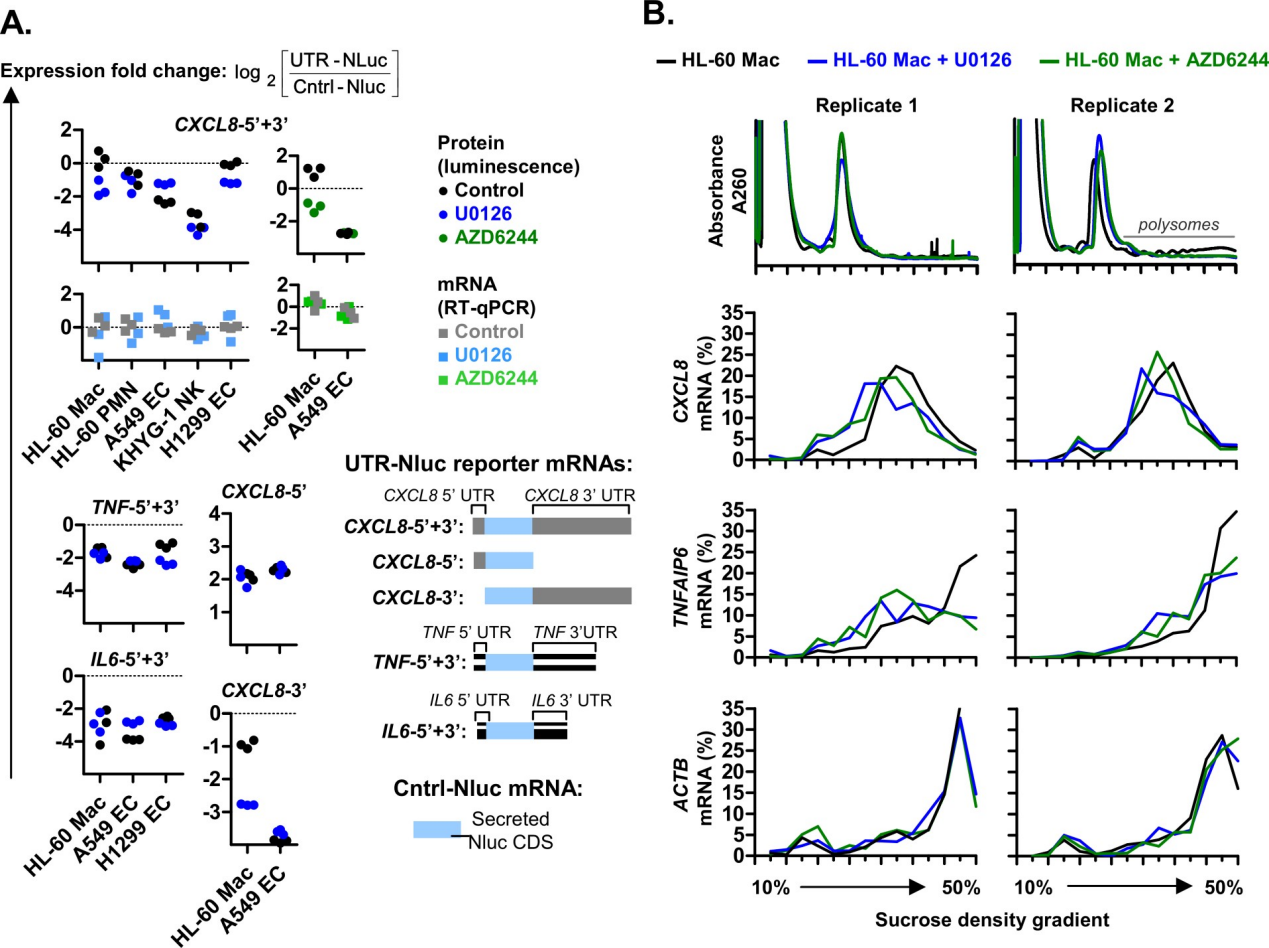
The inhibition of ERK1/2 signaling reduced *CXCL8* UTR reporter expression in HL-60 macrophages. **(A)** Graphical representations of the plasmid-derived UTR-Nluc reporter mRNAs are shown. These plasmids were transfected into parallel cell cultures. Treatments with DMSO solvent control or ERK1/2 signaling inhibitors (10 μM U0126 or 1 μM AZD6244) were performed three hours after UTR-Nluc reporter transfection. The resulting Nluc protein and mRNA expression levels were quantified via luciferase assay and real-time PCR, respectively, after overnight incubation. The ratio of UTR-Nluc over Cntrl-Nluc expression was then determined and presented as log_2_ values. Each graph symbol (squares or circles) is the result of a replicate experiment. Replicate experiments were performed on a different days. **(B)** Polysome profiles of HL-60 Mac after treatment with 10 μM U0126 or 1 μM AZD6244 for 8 hours. The fractionation was performed on a continuous sucrose density gradient (10–50% sucrose). From 14 fractions spanning the entire sucrose gradient, the levels of specific mRNAs (*CXCL8*, *TNFAIP6* and *ACTB*) were quantified via real-time PCR and presented as a percentage of the sum of all fractions. The data for the HL-60 Mac controls were previously shown in Fig 1C. Replicate experiments were performed on different days and the data from 2 replicates are shown.

Next, we elucidated the signaling pathways involved in the elevated expression of *CXCL8*-5’+3’ reporter protein in HL-60 Mac relative to HL-60 PMN, A549 EC and KHYG-1 NK. This cell type-specific expression remained largely unaffected by treatment with the inhibitors of mTOR (Torin-1) (S1C Fig), p38 (SB203580) and JNK (SP600125) (S1D Fig). As controls, the SP600125 treatment used was sufficient to inhibit JNK-mediated phosphorylation of JUN in HL-60 Mac [40] while the SB203580 treatment used was also sufficient to inhibit the transcription of CXCL8 in LPS-activated neutrophils (S1D Fig) [41]. By contrast, the elevated expression of *CXCL8*-5’+3’ protein in HL-60 Mac cells was attenuated when ERK1/2 signaling was inhibited by treatment with U0126 (which inhibits the upstream kinase, MAP2K1/2) or AZD6244 (which directly inhibits ERK1/2) (Fig 6A). The same U0126 treatment also reduced *CXCL8*-3’ reporter protein levels in HL-60 Mac; but did not reduce the expression of the APS-deficient reporters: *CXCL8*-5’, *TNF*-5’+3’ and *IL6*-5’+3’ (Fig 6A).

The inhibition of ERK1/2 signaling in HL-60 Mac cells also reduced the polysome association of endogenous *CXCL8* and *TNFAIP6* relative to control untreated HL-60 Mac cells. This was indicated by a reduction in the percentage of *CXCL8* and *TNFAIP6* mRNA found in the larger polysome fractions (towards the right of the graph) (Fig 6B). The same pattern was not observed for the control mRNA, *ACTB*. These findings suggest that ERK1/2 target proteins may be involved in the cell type-specific expression of APS-positive mRNAs.

### The phosphorylation of rpS6 S235/236 was elevated in HL-60 macrophages and attenuated by ERK1/2 signaling inhibitors

**Fig 7:**
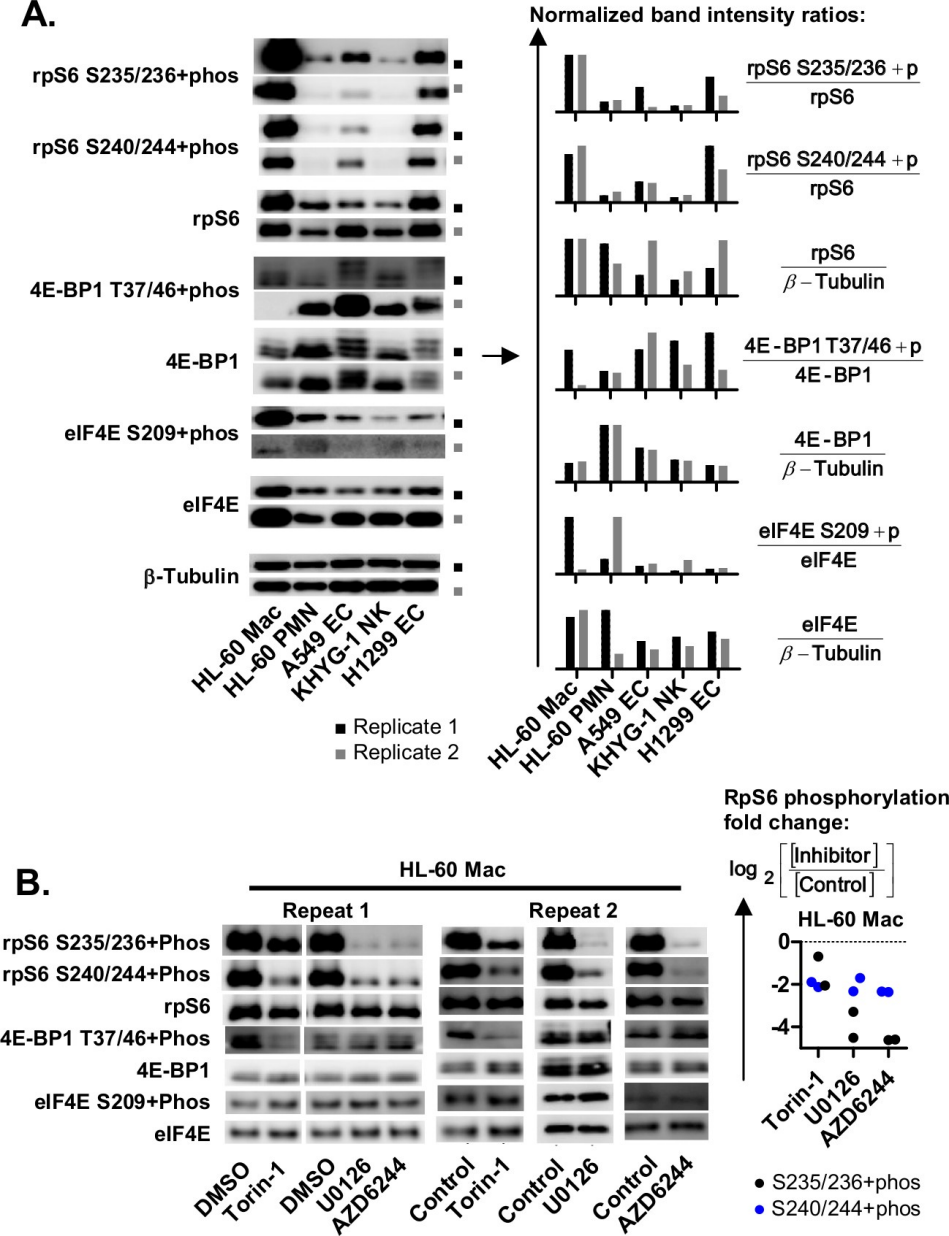
The phosphorylation of rpS6 is elevated in HL-60 macrophages and is sensitive to ERK1/2 signaling inhibition. **(A and B)** Western blots displaying the expression levels of rpS6 S235/236+phos, rpS6 S240/244+phos, total rpS6, 4E-BP1 T37/46+phos, total 4E-BP1, eIF4E S209+phos, total eIF4E and β-Tubulin. The expression levels of these proteins were compared between HL-60 Mac, HL-60 PMN, A549 EC, KHYG-1 NK and H1299 EC cells, in panel A. Comparisons were also made between HL-60 cells treated with the solvent control, ERK1/2 signaling inhibitors (10 μM U0126 or 1 μM AZD6244), and mTOR inhibitor (100 nM Torin-1); in panel B. The band signal intensities were quantified and used to calculate the normalized band intensity ratios as described in the mathematical expressions. In panel B, “rpS6+phos fold change” was calculated based on the mathematical expression shown. [Control] and [Inhibitor] are defined as the rpS6+phos/rpS6 ratios of the control and inhibitor (Torin-1, U0126 and AZD6244) treatment cells, respectively, Experimental replicates were repeated over different days and the data from two repeats are shown.

To identify the ERK1/2 target proteins involved, we examined known downstream targets of ERK1/2 such as rpS6, 4E-BP1 and eIF4E (Fig 7A). Since the protein activities were modulated by their phosphorylation states, the ratios of phosphorylated protein over total protein expression (i.e.: [rpS6 S235/236+phos] / [rpS6]) between different cell types were compared. Amongst the ERK1/2 target proteins, the phosphorylation ratio of rpS6 (at S235/236 and S240/244) was elevated in HL-60 Mac relative to HL-60 PMN, A549 EC, KHYG-1 NK and H1299 EC (Fig 7A). The expression of eIF4E was also elevated in HL-60 Mac relative to A549 EC and KHYG-1 NK (Fig 7A).

Next, we determined if these proteins were affected by the inhibition of ERK1/2 signaling (via U0126 and AZD6244) or mTOR signaling (via Torin-1). Previous studies have found that ERK1/2 signaling induces the phosphorylation of rpS6 at S235/236 [42] while Akt-mTOR signaling induces the phosphorylation of rpS6 at S235/236 and S240/244 [11,43]. Here, the inhibition of ERK1/2 signaling via U0126 or AZD6244 treatment led to 8- to 16-fold reductions in rpS6 S235/236 phosphorylation. The inhibition of mTOR signaling inhibition via Torin-1 treatment led to a much smaller 2- to 4-fold reduction. The smaller effect of Torin-1 on rpS6 S235/236 phosphorylation was unlikely to be due to a lack of Torin-1 activity since the inhibitor almost completely abolished the phosphorylation of 4E-BP1 at T37/46, an effect that was previously described [44]. The phosphorylation at S240/244 on the other hand, was equally sensitive to U0126, AZD6244 and Torin-1 treatment, with a 4-fold reduction across all inhibitors. From these observations, we made three inferences. First, rpS6 S235/236 phosphorylation in HL-60 Mac was predominantly mediated by ERK1/2 signaling. Second, ERK1/2 signaling inhibition may attenuate the expression of APS-positive mRNAs by blocking the phosphorylation of rpS6 at S235/236. Third, ERK1/2 signaling inhibition did not act via the blocking of rpS6 S240/244 phosphorylation since mTOR signaling inhibition also blocked rpS6 S240/244 phosphorylation to a similar degree but did not attenuate *CXCL8*+5’+3’ expression.

### Increasing the availability of eIF4E did not enhance APS-modulated expression

Unlike rpS6, the expression and phosphorylation of eIF4E was not affected by treatment with U0126, AZD6244 or Torin-1 (Fig 7B). This suggests that ERK1/2 inhibitors do not act via eIF4E to attenuate the cell type-specific expression observed. Since the availability of eIF4E to form the translation initiation complex has also been found to modulate the rate of mRNA translation [7–10], further investigations were performed to either confirm or rule out its role. We expressed the 4E-BP1-4A mutant–a phosphorylation deficient mutant of 4E-BP1 which constitutively binds to and inhibits eIF4E activity [4] (S1E Fig). At first glance, this appeared to have reduced *CXCL8*-5’+3’ protein synthesis in HL-60 Mac. However upon closer inspection, it was revealed that multiple proteins (including rpS6 S235/236+phos, rpS6 240/244+phos, total rpS6, eIF4E and μ-tubulin) were up-regulated in HL-60 Mac upon 4E-BP1-4A expression. This made it difficult to narrow down the cause of the reduced *CXCL8*-5’+3’ protein levels. To investigate further, we expressed exogenous eIF4E to alleviate any potential shortage of eIF4E in A549 ECs. However, this did not enhance *CXCL8*-5’+3’ protein synthesis (S1F Fig). This suggests that eIF4E shortage was not a factor at play, which is consistent with our findings on Torin-1 treatment. Treatment with Torin-1 almost completely abolished the phosphorylation of 4E-BP1 at T37/46 in HL-60 Mac cells (Fig 7B) but did not inhibit the expression of *CXCL8*-5’+3’ (S1C Fig). Since the phosphorylation of 4E-BP1 at T37/46 is required for the subsequent “hyper” phosphorylation at S65/T70 which inhibits 4E-BP1 binding to eIF4E [45], this suggests that eIF4E availability was not being reduced by interaction with unphosphorylated 4E-BP1 [46]. Thus, our assays suggest that increasing the availability of eIF4E did not enhance APS-modulated expression.

### The induction of rpS6 S235/236 phosphorylation enhanced the expression of *CXCL8* UTR reporters

**Fig 8:**
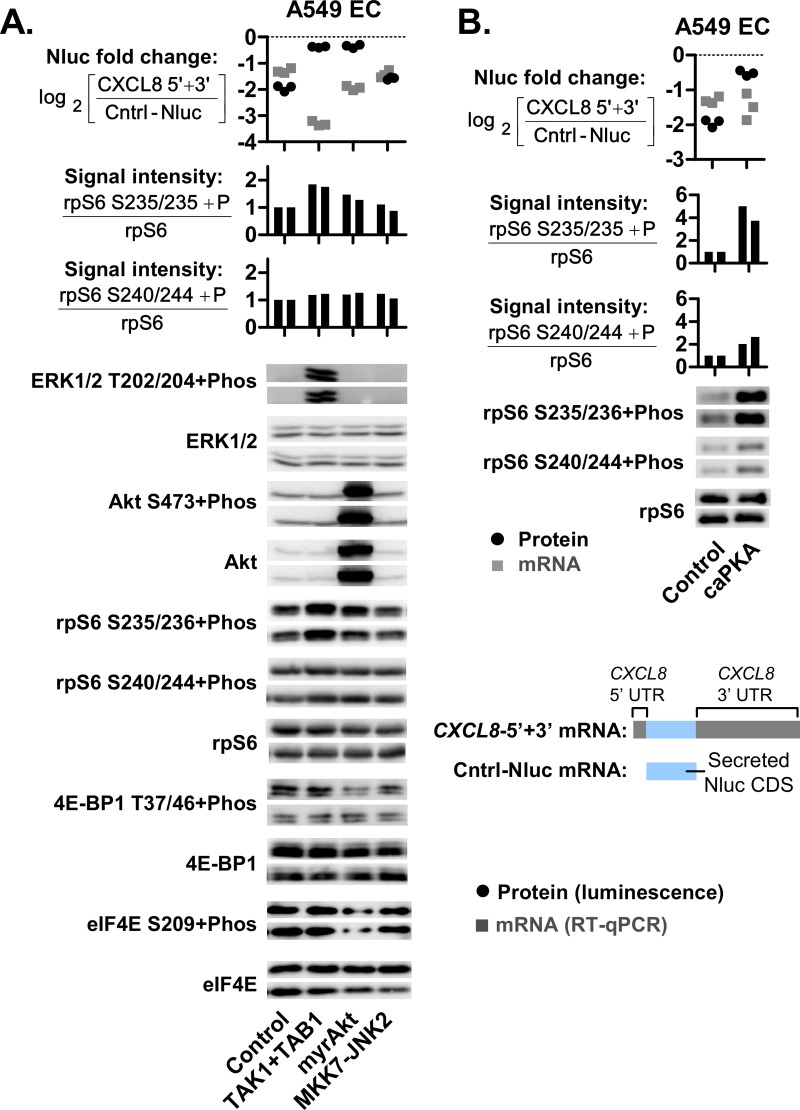
*CXCL8* UTR reporter expression is sensitive to the (rpS6 S235/236+phos)/rpS6 ratio. **(A and B)** Graphical representations of the *CXCL8-*5’+3’ Nluc and Cntrl-Nluc mRNAs are shown. These Nluc reporter plasmids were transfected into parallel cell cultures along with plasmids for the expression of TAK1+TAB1, myrAKT, MKK7-JNK2, caPKA or the control empty pcDNA plasmid. In each of these co-transfection experiments, an equimolar ratio of each plasmid species was used. After overnight incubation, the resulting Nluc protein and mRNA expression levels were quantified via luciferase assay and real-time PCR, respectively. The ratio of UTR-Nluc over Cntrl-Nluc expression was then determined and presented as log_2_ values. Western blots display the expression levels of ERK1/2 T202/204+phos, total ERK1/2, Akt S473+phos, total Akt, rpS6 S235/236+phos, rpS6 S240/244+phos, total rpS6, 4E-BP1 T37/46+phos, total 4E-BP1, eIF4E S209+phos, total eIF4E and β-Tubulin. The band signal intensities were quantified and used to calculate the normalized band intensity ratios as described in the mathematical expressions. The mRNA levels were determined via real-time PCR. Nluc mRNA levels were standardized to the NeoR gene expressed from the pcDNA3.1(+), which is the expression plasmid for the UTR- and Cntrl-Nluc constructs. Each graph symbol (squares or circles) is the result of a replicate experiment. Replicate experiments were performed on different days. For the western blots, two replicates are shown.

To further investigate the role of rpS6, we activated pathways leading to the induction of rpS6 phosphorylation in A549 ECs. There are three known rpS6 kinases that directly phosphorylate rpS6 [11]. The first is p90 ribosomal S6 kinase (Rsk) which is activated by ERK1/2 signaling [42]. We activated this pathway, as indicated by increased ERK1/2 T202/204 phosphorylation, through the co-expression of TAK1 and TAB1 [47] (Fig 8A). The second is p70 S6 protein kinase (S6K1) which is activated by Akt-mTOR signaling [11]. We activated of this pathway, as indicated by increased Akt S473 phosphorylation, through the expression of myristoylated Akt or myrAkt (Fig 8A). Finally, the third known rpS6 kinase is protein kinase A (PKA) [48]. We activated this pathway through the expression of constitutively active PKA (caPKA) (Fig 8B). In A549 EC cells, all three rpS6 kinase pathways induced rpS6 S235/236 phosphorylation and increased *CXCL8*-5’+3’ protein levels (Fig 8A and 8B). The TAK1-ERK1/2 pathway produced a greater degree of rpS6 S235/236 phosphorylation and this coincided with increased *CXCL8*-5’+3’ expression relative to the myrAkt-mTOR pathway. This is consistent with our observations in HL-60 Mac where rpS6 S235/236 phosphorylation was found to be predominantly mediated by ERK1/2 signaling and not Akt-mTOR signaling (Figs 6 and 7). By contrast, *CXCL8*-5’+3’ protein synthesis was not increased when rpS6 S235/236 phosphorylation was not induced, such as during the expression of constitutively active MKK7-JNK (Fig 8A). This is consistent with our earlier findings where the inhibition of JNK activity (via SP600125) did not attenuate *CXCL8*-5’+3’ expression in HL-60 Mac (Fig 6B). In summary, these findings demonstrate that the induction of rpS6 S235/236 phosphorylation enhances *CXCL8*-5’+3’ expression.

### Primary macrophages displayed elevated rpS6 phosphorylation and *CXCL8* translation which were both attenuated by ERK1/2 inhibitors

**Fig 9:**
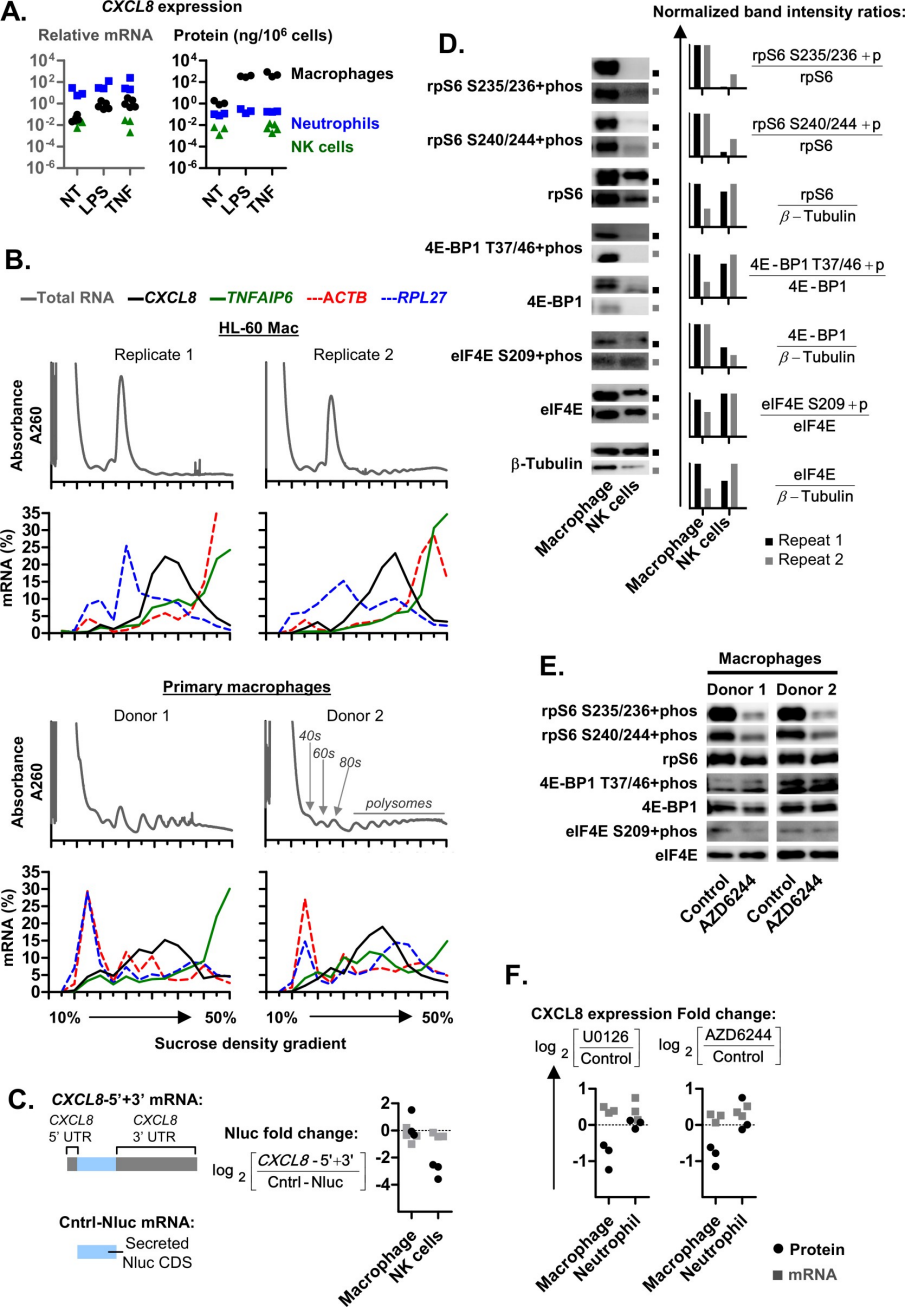
Primary macrophages display an elevated rate CXCL8 translation that is attenuated by ERK1/2 signaling inhibitors. **(A)** CXCL8 protein and mRNA expression levels in primary macrophages, neutrophils and NK cells. Expression was measured at resting state (not treated, NT) or after overnight activation with 100 ng/mL LPS or 10 ng/mL TNF. **(B)** Polysome profiles of primary macrophages and HL-60 Mac were obtained from a continuous sucrose density gradient (10–50% sucrose). From 14 fractions spanning the entire sucrose gradient, the levels of specific mRNAs (*CXCL8*, *TNFAIP6*, *ACTB* and *RPL27*) in each fraction from the sucrose gradients were quantified via real-time PCR and presented as a percentage of the sum of all fractions. The data for the HL-60 Mac repeats were as previously shown in Figs 1C and 5E. **(C)** Graphical representations of the *CXCL8*-5’+3’ UTR-Nluc and Cntrl-Nluc reporter mRNAs are shown. The expression plasmids for these Nluc reporters were transfected into parallel cell cultures. After overnight incubation, the resulting Nluc protein and mRNA expression levels were quantified via luciferase assay and real-time PCR, respectively. The ratio of UTR-Nluc over Cntrl-Nluc expression was then determined and presented as log_2_ value. **(D and E)** Western blots displaying the expression levels of rpS6 S235/236+phos, rpS6 S240/244+phos, total rpS6, 4E-BP1 T37/46+phos, total 4E-BP1, eIF4E S209+phos, total eIF4E and β-Tubulin. The expression levels of these proteins were compared between primary macrophages and NK cells. Comparisons were also made between primary macrophages treated with the solvent control and the ERK1/2 inhibitor, 1 μM AZD6244, The band signal intensities were quantified and used to calculate the normalized band intensity ratios as described in the mathematical expressions. **(F)** The expression ratio of CXCL8 protein and mRNA expression in primary macrophages and neutrophils upon overnight treatment with ERK1/2 signaling inhibitors (10 μM U0126 or 1 μM AZD6244) relative to solvent control cells. **(A to F)** Each graph symbol (squares, triangles or circles) is the result of a replicate experiment. Replicate experiments were performed on different days. Cells from different donors were used for each replicate. For the western blots, two replicates are shown. For panels A and F, CXCL8 protein and mRNA levels were determined via ELISA and real-time PCR, respectively. *CXCL8* mRNA levels are standardized to RPL27 as the internal control genes and are relative to the control NT samples.

Our work on cell lines suggested that *CXCL8* expression was elevated in macrophages via a UTR and ERK1/2-dependent mechanism (Figs 1 to 8). Next, we determined if this mechanism was active in primary macrophages. We found that relative to primary neutrophils and NK cells, primary macrophages secrete disproportionately more CXCL8 protein from less *CXCL8* mRNA (Fig 9A). This matched the trend observed in the cell line counterparts (Fig 1A). Primary macrophages also displayed high levels of *CXCL8* and *TNFAIP* mRNA polysome association, which were comparable to HL-60 Mac (Fig 9B). This occurred despite a reduction in *ACTB* and *RPL27* mRNA polysome association relative to HL-60 Mac. Reduced *ACTB* polysome association was also reported for primary mouse macrophages relative to immortalized mouse macrophage cell lines [49]. Like their HL-60 Mac and KHYG-1 NK cell line counterparts (Fig 2D), CXCL8-5’+3’ protein synthesis was elevated in primary macrophages relative to NK cells (Fig 9C). This suggests that the UTR of *CXCL8* confers elevated translation in primary macrophages relative to NK cells. Additionally, the ratio of phosphorylated rpS6 over total rpS6 was also increased in primary macrophages relative to NK cells (Fig 9D). The elevated rpS6 phosphorylation was attenuated by ERK1/2 inhibitor (AZD6244) treatment (Fig 9E). These results matched their cell line counterparts (Fig 7A and 7B). Finally, primary macrophages treated with the ERK1/2 signaling inhibitors (U0126 and AZD6244) displayed attenuated expression of *CXCL8* at the protein level but not at the mRNA level (Fig 9F). The same inhibitors did not attenuate *CXCL8* expression in primary neutrophils. Taken together, these findings suggest that ERK1/2 signaling induces the phosphorylation of rpS6 in primary macrophages, leading to the elevated expression of *CXCL8* and *TNFAIP6* in these cells.

## Discussion

**Fig 10:**
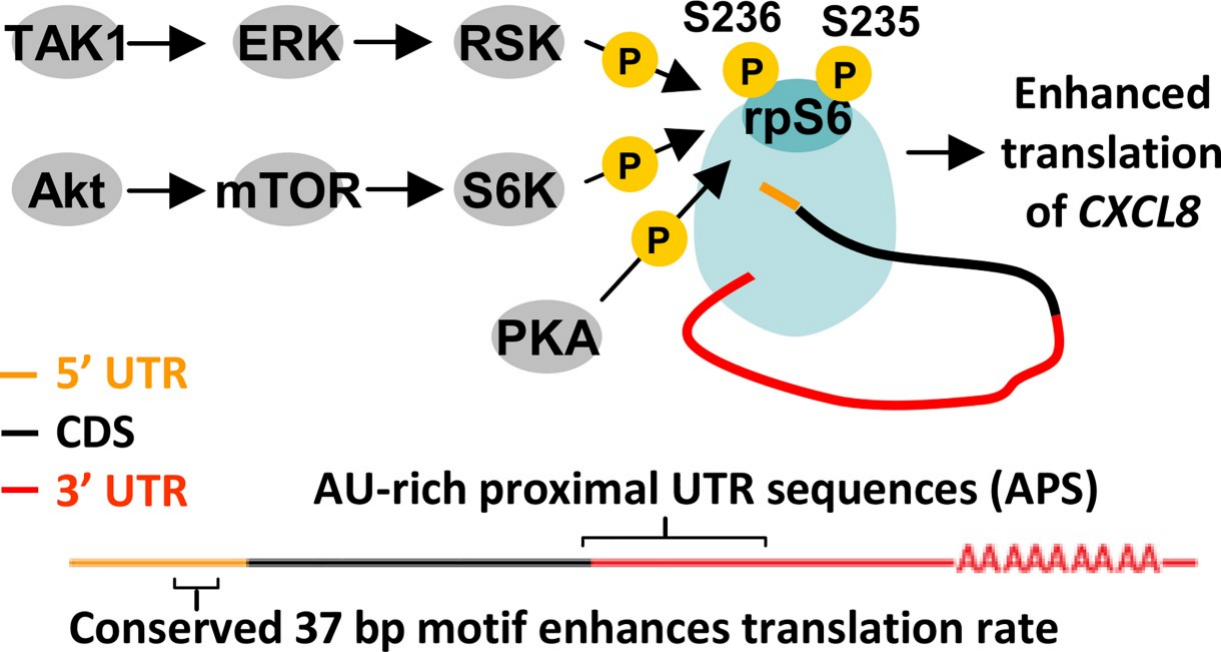
A schematic summary of findings. Ribosomal protein S6 (rpS6) is directly phosphorylated by the kinases Rsk, S6K1 and PKA [11]. Rsk and S6K1 are activated by ERK1/2 and Akt-mTOR signaling respectively. These signaling pathways induce of rpS6 phosphorylation at S235/236 and enhance the translation of CXCL8 mRNA. The enhancement requires: 1) a 3’ UTR that is at least 300 bases; and 2) an AU base content exceeding fifty percent in the first hundred or so bases of the 3’ UTR immediately after the coding sequence, which we refer to as AU-rich proximal RNA cis-regulatory sequences (APS). This translational control mechanism contributes to the elevated CXCL8 protein expression in macrophages. On its own, the 5’ UTR of *CXCL8* is a potent translation enhancer.

Hitherto, the role of rpS6 phosphorylation has remained unclear as it did not appear to affect global mRNA translation rates [15]. Here, we demonstrate that the induction of rpS6 phosphorylation (at S235/236) selectively enhances the translation of certain mRNA transcripts that contain novel AU-rich proximal cis-regulatory UTR sequences (APS) [Summarized in Fig 10]. In primary macrophages, ERK1/2 signaling induces the phosphorylation of rpS6 leading to the elevated translation of *CXCL8* and *TNFAIP6*. The location of the APS-motif, on the 3’ UTR, is distinct from known motifs found exclusively in the 5’ UTR [4–6,8,9].

Like previous work on *CXCL8* [19–26], our studies were conducted on primary human cells and cell line models. Studies on *CXCL8* expression could not use the mouse model since the homolog of *CXCL8* is deleted in mice [16]. This also meant that we could not use phospho-deficient rpS6 transgenic mice [14], although it would have been a powerful tool to investigate the role of rpS6 phosphorylation. To study the effect of rpS6 phosphorylation, we inhibited multiple pathways upstream of rpS6 phosphorylation. The mTOR inhibitor (Torin-1) only partially attenuated rpS6 S235/236 phosphorylation (Fig 7B) and did not reduce the translation of APS-positive *CXCL8-5’+3’* in HL-60 macrophages (S1C Fig). By contrast, when rpS6 S235/236 phosphorylation was more completely attenuated by the ERK1/2 signaling inhibitors (AZD6244 and U0126) (Figs 7B and 9E); the translation of APS-positive *CXCL8-5’+3’*, *CXCL8-3’*, *CXCL8* and *TNFAIP6* reporters in macrophages was reduced (Figs 6 and 9F). The effect of ERK1/2 signaling inhibition was cross-validated by two different inhibitors since both inhibitors are unlikely to share the same off-target effects, such as the activation of AMPK by U0126 [50]. All three inhibitors–Torin1, U0126 and AZD6244–also reduced rpS6 S240/244 phosphorylation to a similar degree (Fig 7B). This suggests that the U0126 and AZD6244 treatments did not act via the inhibition of rpS6 S240/244 phosphorylation to attenuate *CXCL8*-5’+3’ expression in HL-60 Mac (Fig 6A). If that were the case, the Torin-1 treatment should have been just as potent in attenuating reporter expression (S1C Fig). From these findings, we deduced that the elevated rpS6 S235/236 phosphorylation observed in HL-60 Mac was predominantly mediated by ERK1/2 signaling and that ERK1/2 inhibitors may attenuate the expression of APS-positive mRNAs by blocking this pathway.

In addition to kinase inhibitors, the expression of exogenous wildtype and mutant proteins was also employed to study the role of rpS6 phosphorylation. The expression of constitutively active TAK1, Akt or PKA led to increased expression of the APS-positive reporter, *CXCL8*-5’+3’. This was likely due to the increased rpS6 S235/236 phosphorylation observed (Fig 8A and 8B). To the best of our knowledge, rpS6 is the only known common downstream target of the TAK1, Akt and PKA pathways. TAK1 signaling activates Rsk while Akt signaling activates S6K1. PKA, Rsk and S6K1 are the three currently known rpS6 kinases that directly phosphorylate rpS6 at S235/236 [11]. Even though PKA is not known to phosphorylate rpS6 at S240/244, a small increase was observed in the caPKA transfected cells (Fig 8B). The increase was however, still at least 5-fold lower than that observed for 235/236. Taken together, these findings support the hypothesis that the induction of rpS6 S235/236 phosphorylation enhances the translation of APS-positive mRNAs.

Multiple mechanisms may contribute to the elevated level of CXCL8 protein secreted from macrophages. The 3’ UTR sequences have also been found to modulate the rate of mRNA transcription, mRNA stability and pre-recruitment of the signal recognition particle to the ribosome for secreted proteins [51–53]. Alterations in the rate of secretion could also occur as a result of changes in the activity of the eIF4E kinase, MNK1 [54]. Nevertheless, the rate of secretion is unlikely to be involved in the elevated cytoplasmic expression of *CXCL8*-5’+3’(-Sec) protein observed in HL-60 Mac relative to A549 EC (Fig 2G). Another possible mechanism may involve the ARE-motifs on the 3’ UTR of *CXCL8* which reportedly accelerate mRNA degradation [34–36]. This may explain the reduced expression levels of *CXCL8*-full mRNA relative to *CXCL8*-CDS mRNA (Fig 2A and 2B). However, the elevated expression of *CXCL8*-5’+3’ in macrophages was retained even after the deletion of these ARE motifs (Fig 4A), which suggests that these ARE motifs were not responsible for the cell type-specific expression. Additionally, increased transcription or mRNA stability would lead to increased mRNA levels which, in this case, did not account for the level of secreted CXCL8 protein observed in macrophages that was elevated relative to *CXCL8* mRNA levels (Figs 1A, 2 and 9A). Thus, the elevated *CXCL8* protein secretion in macrophages is most simply explained by an increase in the rate of *CXCL8* translation, which was corroborated by the polysome profiles of *CXCL8* in macrophages (Figs 1C and 9B).

Further studies are required to fully elucidate this novel mechanism of translational regulation. While it is clear that the mechanism involves: 1) a 3’ UTR that is at least a few hundred bases; and 2) an APS element; there may be other subtle requirements that await further discovery. The sequence feature in the *CXCL8* 5’ UTR that is responsible for its ability to enhance translation in macrophages when paired with a 3’ UTR (Fig 5C) also warrants further investigation. The 3’ UTR of CXCL8 in particular, is also known to be recognized by miRNA-93, leading to the downregulation of CXCL8 mRNA and protein levels [55]. However, since various deletion mutants of the CXCL8 3’ UTR retained the same pattern of elevated expression in macrophages (Fig 4B), it is highly unlikely that the same level of miRNA activity was exerted across all these deletion mutations. Additionally, it is likely that other pathways besides rpS6 phosphorylation are involved in the regulation of APS-positive mRNA expression. Certain observations point to this. For example, while the inhibition of ERK1/2 (via U0126) attenuated rpS6 S235/236 phosphorylation (Fig 7B), it did not reduce *CXCL8*-5’+3’ protein levels in HL-60 Mac to the levels observed in KHYG-1 NK cells (Fig 6A).

The APS-motif was elucidated in mutation assays with heterologous vector-derived CXCL8 and UTR-Nluc reporter transcripts. Vector-derived transcripts are widely employed to evaluate UTR activity and have so far been consistent with native mRNAs [5,6,8–10,51]. For example, both the endogenous mRNAs and vector-derived reporters that contain 5’ UTR CERT domains display selective translational repression in eIF4E haplo-insufficient mouse cells [5]. In another study, the deletion of the 3’ UTRs of endogenous *CXCL1*, *CXCL6* and *CXCL8*; led to increased mRNA stability which confirmed earlier findings based on heterologous reporter assays [51]. The deletion also led to an unexpected reduction in mRNA transcription leading to a decrease in mRNA levels despite increased mRNA stability. Thus, while the deletion of endogenous UTR sequences may lead to unintended effects on transcription, vector-derived transcripts have consistently replicated native UTR activity. Indeed, for this study, we found that the cell type-specific translation rate of endogenous CXCL8 matched that of vector-derived *CXCL8*-full mRNAs (Fig 2A) and *CXCL8*-5’+3’ UTR-Nluc reporters (Fig 2D)

Future research to establish the significance of APS in regulating gene expression and its evolutionary conservation is warranted. In addition to *CXCL8* and *TNFAIP6*, other APS-positive mRNAs may exist. An analysis of the proximal AU content in the 3’ UTR of common cytokine signaling genes revealed the presence of potential APS in the mRNAs of *TNFAIP6*, *IFNG* and *IL2* (Fig 5D) but only the *TNFAIP6* mRNA was expressed at sufficiently high levels for polysome profilling (Figs 5E, 6B and 9B). APS-positive mRNAs may form a highly selective subset of genes as the distribution of 3’ UTR AU content in human genes appears to be skewed towards a low AU content [56]. The identification and characterization of genes that contain APS motifs may be an important area for future studies as this novel mechanism of translational control may regulate other genes that impact disease processes.

In conclusion, we propose that rpS6 phosphorylation at S235/236 selectively enhances the translation of mRNAs that contain APS, a potentially novel RNA *cis*-regulatory element. These mRNAs possess: 1) a 3’ UTR of at least three hundred bases; and 2) an AU base content that exceeds fifty percent in the first hundred or so bases of the 3’ UTR immediately after the stop codon. In primary macrophages, the ERK1/2 pathway induces the phosphorylation of rpS6 leading to the elevated expression of *CXCL8* and *TNFAIP6*. This novel translational control mechanism allows macrophages to act as the primary producer of CXCL8 protein despite expressing less CXCL8 mRNA than cells such as neutrophils (Fig 9A). Through the secretion of CXCL8, macrophages recruit and activate other immune cells such as neutrophils [19], monocytes and lymphocytes [20] to respond to infection, disease or injury. APS-modulated translation may also be aberrantly upregulated in a subset of tumour cells, leading to the overexpression of pro-oncogenic genes such as CXCL8. Modulating the rate of APS-positive mRNA translation, such as CXCL8, may be a novel strategy to treat diseases [57].On its own, the 5’ UTR of *CXCL8* is a potent translation enhancer. The truncated 5’ UTR sequence (75 bp) could potentially be used to enhance the expression of therapeutic proteins.

## Materials and methods

### Ethics statement

This work was approved by the Institutional Review Board (IRB), NUS-IRB B-14-063E, National University of Singapore (NUS).

### Expression vectors

The CXCL8, Nluc and mCherry reporter vectors were generated as described in S1 Table. Positions 79 to 153 of NM_000584 was inserted immediately upstream of pNFAT Fluc (Addgene plasmid #10959 [58]) to form pNFAT 53’ Fluc. PCW57.1-4EBP1_4xAla (from Prof David Sabatini, Addgene plasmid # 38240 [4]); NFAT luciferase reporter (from Toren Finkel, Addgene plasmid #10959 [58]); pCNA-TAK1-WT, pCMV5-TAB1-FLAG, pCDNA-myr-AKT, pCDNA JNK/SAPK-MKK7 and pCMV-PKAca.

### Primary cell isolation

Human peripheral blood monocytes, neutrophils and NK cells were isolated from healthy male adult donor aphaeresis cones (National University Hospital, Blood Donation Centre, Singapore). This work was approved by the Institutional Review Board (IRB), NUS-IRB B-14-063E, National University of Singapore (NUS). Monocytes and NK cells were isolated as described previously [59]. Briefly, the buffy coat, which contained citrate-phosphate-dextrose (CPD) as anticoagulant, was diluted four times with PBS containing 2% FBS and 1 mM EDTA, and the mononuclear fraction was obtained via density gradient centrifugation with Ficoll-Paque Premium 1.073 (GE Healthcare). From the mononuclear fraction, the monocyte and NK cell populations were enriched with the Human Monocyte Enrichment Kit and Human NK Cell Enrichment Kit (Stemcell), respectively. For neutrophil isolations, buffy coats were diluted using Hank’s Balanced Salt Solution (HBSS; Life Technologies). Red blood cells were sedimented by gravity using Hetasep solution (Stemcell). Leukocytes were harvested and neutrophils were isolated using Human Neutrophil Enrichment kit (Stemcell).

### Cell culture

Human macrophages, peripheral blood monocytes, neutrophils, NK cells, HL-60 cells, A549 cells, NCI-H1299 and KHYG-1 cells were cultured in RPMI (with 10% FBS and 1% v/v penicillin and streptomycin) at 37 ^o^C. KHYG-1 cell media was supplemented with 10 ng/mL IL-2. All cell lines were routinely tested to ensure mycoplasma-free cultures.

### Cell differentiation

Enriched human peripheral blood monocytes, at a cell density of 2 × 10^6^/ml, were differentiated into macrophages by culture at 37 ^o^C for 7 days in RPMI media containing 10% FBS and 1% v/v penicillin and streptomycin, and supplemented with 50 ng/ml M-CSF. HL-60 cells were differentiated into a macrophage-like phenotype (HL-60 macrophage) by plating the cells at a density of 2 x 10^6^ cells/mL and culturing in media supplemented with 50 nM phorbol 12-myristate 13-acetate (PMA) for a day followed by another day in PMA-free media. HL-60 cells were differentiated into a polymorphonuclear neutrophil-like phenotype (HL-60 PMN) by plating the cells at a density of 1 x 10^6^ cells/mL and culturing in media supplemented with 1.2% (v/v) dimethyl sulfoxide (DMSO) for 5 days.

### Flow cytometry analysis of enriched leukocyte preparations

Primary cell isolations were routinely analyzed by flow cytometry, with mouse monoclonal antibodies from eBioscience. Preparations were typically more than: 85% CD14^+^CD16^-^ for monocytes; 99% CD11b^+^CD14^+^ for macrophages; 95% CD66b^+^CD16^+^ for neutrophils; and 85% CD3^+^CD56^+^ for NK cells.

### Polysome profiling

Cells were treated at a final concentration of 10 μM U0126 (Cell Signaling) or 1 μM AZD6244 (Selleckchem) for 8 hours before samples were collected. Polysome profiling was performed as described previously [60]. Briefly, cells were preincubated with cycloheximide (100 μg/mL, Sigma) for 15 min, and cytoplasmic lysates were prepared and fractionated by ultracentrifugation through 15%–50% linear sucrose gradients; 14 fractions were collected, and RNA extracted from each fraction was used for quantitative real-time PCR analysis.

### Cell treatments, Nluc and ELISA assays

Immediately prior to transfection or treatment, cells were plated at a density of: 5 x 10^6^ cells/ml for neutrophils; 2 x 10^6^ cells/ml for NK cells and HL-60 PMN; and 1 x 10^6^ cells/ml for macrophages and KHYG-1 cells. A549 cells were plated the day before at a density of 10^6^ cells/mL. For HL-60 macrophages, undifferentiated HL-60 cells were plated at a density of 2 x 10^6^ cells/mL in media supplemented with 50 nM PMA for two days. Treatments and transfections were performed in PMA-free media. Macrophage, HL-60 macrophages, NK cell, HL-60 PMN and KHYG-1 transfections were performed with the Neon Transfection System (Invitrogen); while A549 and H1299 transfections were performed with ViaFect Transfection Reagent (Promega). Two hours after transfection or in non-transfection controls, cells were treated at a final concentration of 100 ng/mL *Escherichia coli* 055:B5 LPS (Sigma), 10 ng/mL TNF (Gibco), 10 μM U0126 (Cell Signaling), 1 μM AZD6244 (Selleckchem), 10 μM SB203580 (Cell Signaling), 50 μM SP600125 (Cell Signaling) or 200 nM Torin-1 (MedChem Express) overnight before samples were collected. Secreted cytokines were measured using Human CXCL8 and IL6 ELISAs (BD OptEIA). Nluc was assayed with Nano-Glo Luciferase Assay System (Promega). Firefly luciferase (Fluc) was assayed with Luciferase Assay System (Promega) using. Luminescence was measured with GLOMAX 20/20 luminometer (Promega).

### Real-time quantitative PCR analysis

Real-time PCR was performed as previously described [61]. RNA was extracted in the presence of 4 μg glycogen (Ambion) using two rounds of Trizol Reagent (Ambion) to obtain a DNA-free sample. Purified RNAs were stored at -80 ^o^C for less than a week before reverse transcription. RNA (0.5 μg via nanodrop) was primed with random hexamers and reverse transcribed with the SuperScript III First-Strand Synthesis System (Invitrogen). The resulting cDNA was stored in TE buffer at -20 ^o^C. The cDNA was analyzed by real-time qPCR using the GoTaq qPCR Master Mix (Promega) and a LightCycler 480 (Roche). qPCR experiments including primer design and efficiency tests was carried out according to the Minimum Information for Publication of Quantitative Real-Time PCR Experiments (MIQE) guidelines [62]. With *RPL27* [63] (123 bp amplicon on NM_000988; forward primer:ATCGCCAAGAGATCAAAGATAA; reverse primer:TCTGAAGACATCCTTATTGACG) and *NeoR* (84 bp amplicon on NeoR marker of pcDNA3.1; forward primer: CAAGATGGATTGCACGCAGG; reverse primer: GCAGCCGATTGTCTGTTGTG) as the reference genes, we calculated the mean fold change of *CXCL8* (132 bp amplicon on NM_000594.2; forward primer: TGTGAAGGTGCAGTTTTGCCAAGG; reverse primer: GTTGGCGCAGTGTGGTCCACTC) and *Nluc* (117 bp amplicon on CDS of secNluc (Promega); forward primer: GTGTCCGTAACTCCGATCCA; reverse primer: TTCGATCTGGCCCATTTGGT). The expression levels relative to the control was calculated using the 2^-**ΔΔ**CT^ method. *RPL27* has been previously validated to be stably expressed across a multitude of different cell types and experimental conditions [63]. Polysome profiling used the same RPL27 and CXCL8 primers and included the TNFAIP6 (212 bp amplicon on NM_007115.3; forward primer: TGCTGGATGGATGGCTAAGG; reverse primer: ACTCATTTGGGAAGCCTGGAG) and ACTB (121 bp amplicon on NM_001101.5; forward primer: GTCATTCCAAATATGAGATGCGT; reverse primer: GCTATCACCTCCCCTGTGTG) primers. DNA contamination was routinely assessed via qPCR of negative control reverse transcription reactions which lacked reverse transcriptase (cycle thresholds above 35).

### Western blot analysis

Total cell lysates were resolved on a denaturing SDS PAGE gel (12%) and transferred onto PVDF membranes via the Trans-Blot Turbo Transfer System (Biorad). These were then probed with antibodies (all from Cell Signaling) against rpS6 S235/236+phos (#4858), rpS6 S240/244+phos (#5364), total rpS6 (#2217), 4E-BP1 T37/46+phos (#2855), total 4E-BP1 (#9644), eIF4E S209+phos (#9741), total eIF4E (#2067), JUN S73+phos (#3270), total JUN (#9165), ERK1/2 T202/204+phos (#4370), total ERK1/2 (#4695), Akt S473+phos (#4060), total Akt (#9272), and β-Tubulin (#2067). These primary antibodies were then probed with the respective HRP-conjugated secondary antibodies. Western blot chemiluminescent signals were captured with an ImageQuant LAS 4000 mini (GE Healthcare). Using ImageQuant TL 7.0 (GE Healthcare), the band signal intensities of each sample were background subtracted (minimum profile) and normalized to control samples for the inhibitor treatments or the highest intensity cell sample between different cell types.

### Bioinformatics analysis

The RefSeg mRNA sequences for human (NM_000594.2), horse (NM_001083951.2), pig (NM_213867.1), cattle (NM_173925.2) and sheep (NM_001009401.2). RNA sequence motifs were predicted with RegRNA2.0 [39].

## Supporting information

S1 Table Generation of expression vectors for CXCL8, Nluc and mCherry coding sequences with and without UTR fusions. (TIF)

S1 Fig **(A)** Fluorescence micrographs showing GFP expression in HL-60 Mac, A549 EC and KHYG-1 NK a day after transfection. **(B)** Phase contrast micrograph of HL-60 Mac morphology. **(C, D, E and F)** UTR-Nluc reporter plasmids were transfected into parallel cell cultures. Graphical representations of the plasmid-derived UTR-Nluc reporter mRNAs are shown in Fig 6A. The resulting Nluc protein and mRNA expression levels were quantified via luciferase assay and real-time PCR, respectively, after overnight incubation. The ratio of UTR-Nluc over Cntrl-Nluc expression was then determined and presented as log_2_ values. Each graph symbol (squares or circles) is the result of a replicate experiment. Replicate experiments were performed on different days. For the western blots, the data for two replicates are shown. In panels C and D, treatments with DMSO solvent control, p38 (10 μM SB203580), JNK (50 μM SP600125) and mTOR inhibitor (100 nM Torin-1) were performed three hours after UTR-Nluc reporter transfection. In panel D, the positive controls for SB203980 and SP600125 activity are shown to the right of their respective Nluc fold change graphs. CXCL8 mRNA levels in neutrophils after overnight treatment with 100 ng/mL LPS or 10 μM SB203580 or both. *CXCL8* mRNA levels were determined via real-time PCR and presented as the ratio of *CXCL8* divided by the internal control gene, *RPL27*. Western blots of total JUN and JUN S73+Phos protein expression in HL-60 Mac that were treated with 50 μM SP600125 or a solvent control for 8 hours. In panels E and F, UTR- Nluc reporter plasmids were transfected into parallel cell cultures along with plasmids for the expression of 4E-BP1 4A, eIF4E or the control empty pcDNA plasmid. In each of these co-transfection experiments, an equimolar ratio of each plasmid species was used. Western blots display the expression levels of rpS6 S235/236+phos, rpS6 S240/244+phos, total rpS6, total 4E-BP1, total eIF4E and μ-Tubulin.
(TIF)

S1 File Underlying numerical data for all graphs found in Fig 1 to Fig 10. (XLS)
