# Supplementary figures and images for: Novel AU-rich proximal UTR sequences (APS) enhance CXCL8 synthesis upon the induction of rpS6 phosphorylation
Source: PLoS Genet. 2019 Apr 10;15(4):e1008077. doi: 10.1371/journal.pgen.1008077 (PMC6476525; doi:10.1371/journal.pgen.1008077)

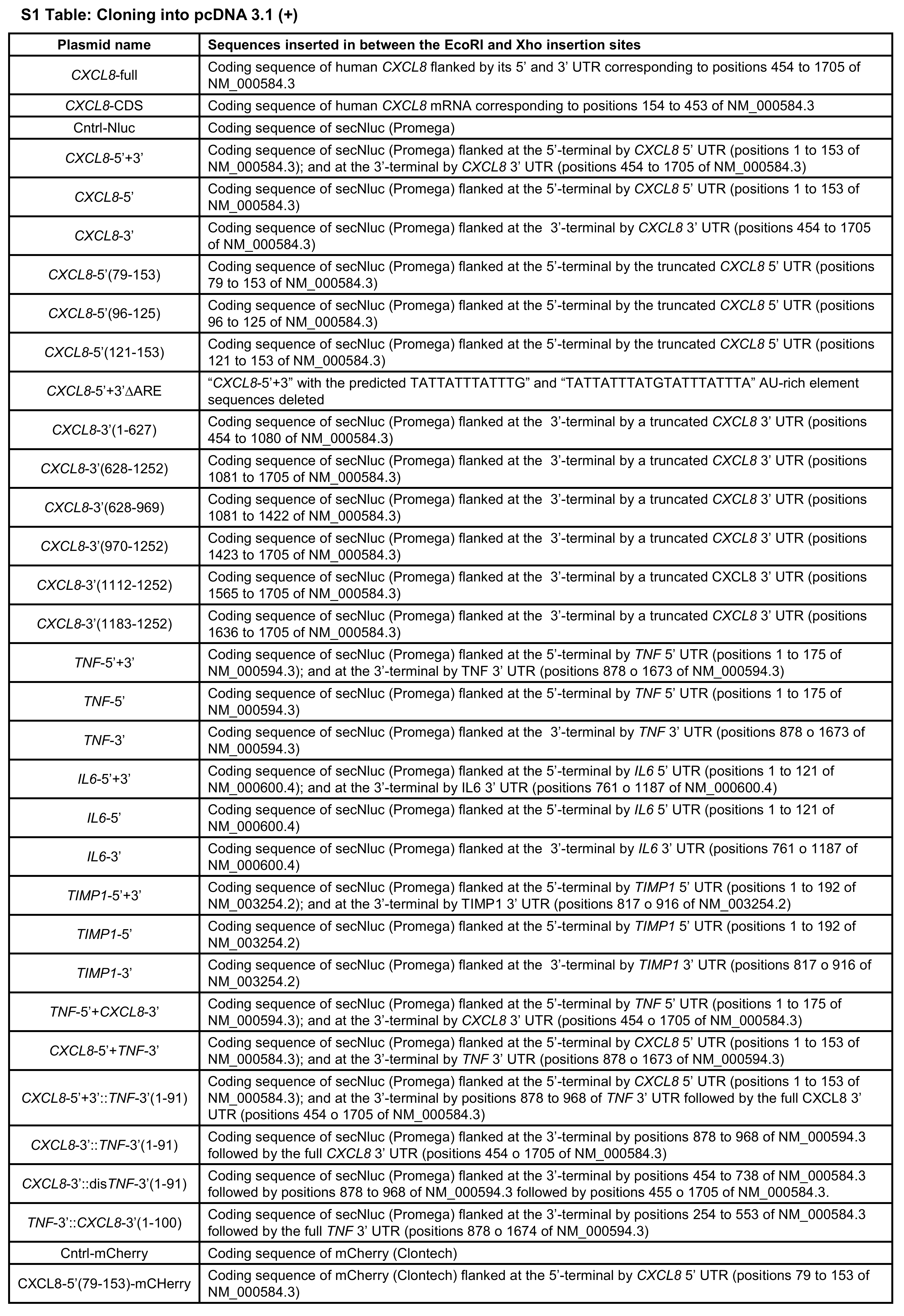

Supplement: S1 Table — (TIF) [file pgen.1008077.s001.tif]

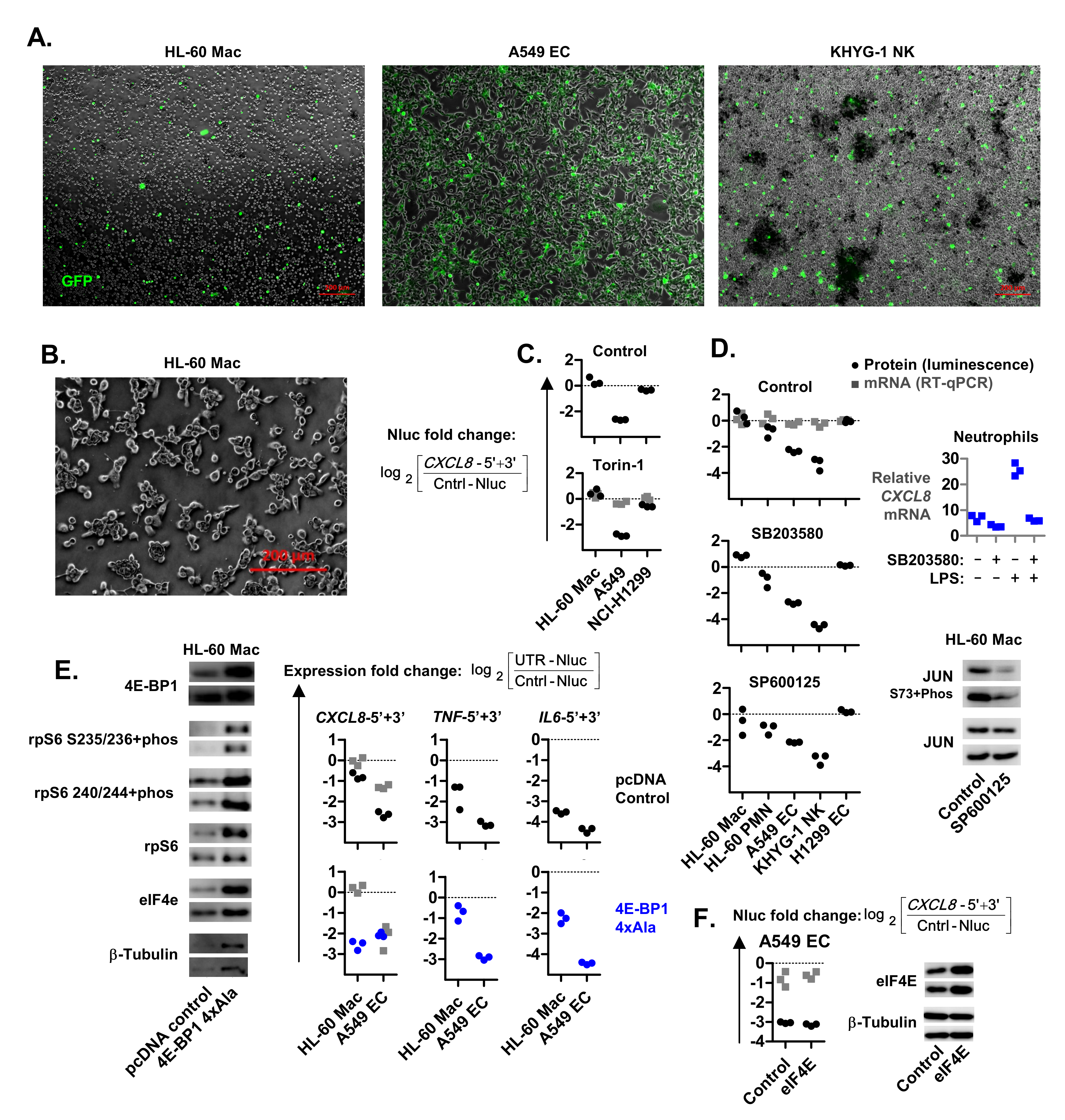

Supplement: S1 Fig — (A) Fluorescence micrographs showing GFP expression in HL-60 Mac, A549 EC and KHYG-1 NK a day after transfection. (B) Phase contrast micrograph of HL-60 Mac morphology. (C, D, E and F) UTR-Nluc reporter plasmids were transfected into parallel cell cultures. Graphical representations of the plasmid-derived UTR-Nluc reporter mRNAs are shown in Fig 6A. The resulting Nluc protein and mRNA expression levels were quantified via luciferase assay and real-time PCR, respectively, after overnight incubation. The ratio of UTR-Nluc over Cntrl-Nluc expression was then determined and presented as log2 values. Each graph symbol (squares or circles) is the result of a replicate experiment. Replicate experiments were performed on different days. For the western blots, the data for two replicates are shown. In panels C and D, treatments with DMSO solvent control, p38 (10 μM SB203580), JNK (50 μM SP600125) and mTOR inhibitor (100 nM Torin-1) were performed three hours after UTR-Nluc reporter transfection. In panel D, the positive controls for SB203980 and SP600125 activity are shown to the right of their respective Nluc fold change graphs. CXCL8 mRNA levels in neutrophils after overnight treatment with 100 ng/mL LPS or 10 μM SB203580 or both. CXCL8 mRNA levels were determined via real-time PCR and presented as the ratio of CXCL8 divided by the internal control gene, RPL27. Western blots of total JUN and JUN S73+Phos protein expression in HL-60 Mac that were treated with 50 μM SP600125 or a solvent control for 8 hours. In panels E and F, UTR- Nluc reporter plasmids were transfected into parallel cell cultures along with plasmids for the expression of 4E-BP1 4A, eIF4E or the control empty pcDNA plasmid. In each of these co-transfection experiments, an equimolar ratio of each plasmid species was used. Western blots display the expression levels of rpS6 S235/236+phos, rpS6 S240/244+phos, total rpS6, total 4E-BP1, total eIF4E and μ-Tubulin. (TIF) [file pgen.1008077.s002.tif]
